# Acidophilic methanotrophs: Occurrence, diversity, and possible bioremediation applications

**DOI:** 10.1111/1758-2229.13156

**Published:** 2023-04-11

**Authors:** Myung Hwangbo, Yiru Shao, Paul B. Hatzinger, Kung‐Hui Chu

**Affiliations:** ^1^ Zachry Department of Civil and Environmental Engineering Texas A&M University College Station Texas USA; ^2^ Aptim Federal Services, LLC 17 Princess Road Lawrenceville New Jersey USA

## Abstract

Methanotrophs have been identified and isolated from acidic environments such as wetlands, acidic soils, peat bogs, and groundwater aquifers. Due to their methane (CH_4_) utilization as a carbon and energy source, acidophilic methanotrophs are important in controlling the release of atmospheric CH_4_, an important greenhouse gas, from acidic wetlands and other environments. Methanotrophs have also played an important role in the biodegradation and bioremediation of a variety of pollutants including chlorinated volatile organic compounds (CVOCs) using CH_4_ monooxygenases via a process known as cometabolism. Under neutral pH conditions, anaerobic bioremediation via carbon source addition is a commonly used and highly effective approach to treat CVOCs in groundwater. However, complete dechlorination of CVOCs is typically inhibited at low pH. Acidophilic methanotrophs have recently been observed to degrade a range of CVOCs at pH < 5.5, suggesting that cometabolic treatment may be an option for CVOCs and other contaminants in acidic aquifers. This paper provides an overview of the occurrence, diversity, and physiological activities of methanotrophs in acidic environments and highlights the potential application of these organisms for enhancing contaminant biodegradation and bioremediation.

## INTRODUCTION

Methanotrophs, which exist in various environments, are bacteria that grow on methane (CH_4_) as a sole source of carbon and energy (Cai et al., 2016; Guerrero‐Cruz et al., 2021; Holmes et al., 1999). Methanotrophs oxidize CH_4_ to methanol (CH_3_OH), formaldehyde (HCHO), formic acid (HCOOH), and ultimately carbon dioxide (CO_2_). CH_4_ monooxygenase (MMO), which is critical in C1 metabolism, is responsible for the first step of CH_4_ oxidation to CH_3_OH (Gesicka et al., 2021; Guerrero‐Cruz et al., 2021; Hanson, 1996). There are two general types of MMO, particulate CH_4_ monooxygenase (pMMO), which is an intracytoplasmic membrane‐bound copper‐containing enzyme, and soluble CH_4_ monooxygenase (sMMO), which is an iron‐containing cytoplasmic enzyme (Lawton & Rosenzweig, 2016; Lee et al., 2020). Most methanotrophs have pMMO only, some contain both pMMO and sMMO, and very few contain only sMMO.

Methanotrophs have been studied in the field of pollutant biodegradation because they can oxidize environmental contaminants including many different chlorinated volatile organic compounds (CVOCs) using MMO (Chu & Alvarez‐Cohen, 1996; Chu & Alvarez‐Cohen, 1998; Chu & Alvarez‐Cohen, 1999; Oldenhuis et al., 1991; Semrau, 2011). Among the CVOCs oxidized by MMO, trichloroethylene (TCE) is particularly important in that it is widely distributed in groundwater aquifers, and causes negative effects on the immune and central nervous systems as well as being a suspected carcinogen (EPA, 2016). Common anaerobic degradation products of TCE, such as vinyl chloride (VC; a potent carcinogen) and *cis*‐1,2‐dichloroethene (*cis*‐DCE), are also degraded by MMO, as are numerous other halogenated organic compounds (Samin & Janssen, 2012; Schäfer et al., 2003). While methanotrophs have been successfully applied for the bioremediation of CVOC‐contaminated aquifers (e.g. Hazen et al., 1994), most in situ remediation of CVOCs is performed via anaerobic bioremediation using carbon source addition, with or without bioaugmentation with dechlorinating consortia containing *Dehalococcoides* spp., one of two groups of organisms (along with a recently discovered *Dehaligenomonas* spp.) known to be capable of dehalogenating PCE and TCE all the way to ethene (Chen et al., 2022; Stroo & Ward, 2010; Yang et al., 2017). However, anaerobic bioremediation by *Dehalococcoides* spp. is largely ineffective at reducing chlorinated ethenes to ethene in naturally acidic aquifers, because it is typically inhibited at pH < ~5.5 (Eaddy, 2008; Lacroix et al., 2014; Rowlands, 2004; Steffan & Vainberg, 2013; Vainberg et al., 2009; Yang, 2012; Yang et al., 2017). Although much more research is required, acidophilic methanotrophs have recently been observed to degrade a range of CVOCs at pH <5.5, suggesting that cometabolic treatment may be an option for CVOCs and other contaminants in acidic aquifers (Choi et al., 2021; Semrau, 2011; Shao et al., 2019; Szwast, 2021).

For this review, an ‘acidophilic methanotroph’ is defined generally as capable of growth at pH 5 or below, with the understanding that these organisms are not necessarily ‘obligate acidophiles’ that require highly acidic pH to survive, consistent with the definition by Madigan (2018). Acidophilic methanotrophs have recently been identified in a wide variety of acidic environments, including peat bogs, wetlands and lakes, thermal soils and springs, and groundwater aquifers among others. Methanotrophs in acidic wetlands are critically important for the control of atmospheric CH_4_, which is important in the global carbon cycle (Nguyen et al., 2018; Siljanen et al., 2012), because typically more than 90% of CH_4_ produced in wetlands is oxidized by methanotrophs in the surface layers (Oremland & Culbertson, 1992; Siljanen et al., 2012). Moreover, the control of CH_4_ fluxes by acidophilic methanotrophs becomes more and more important due to accelerated soil acidification via anthropogenic activities and climate change (Nguyen et al., 2018).

**FIGURE 1:**
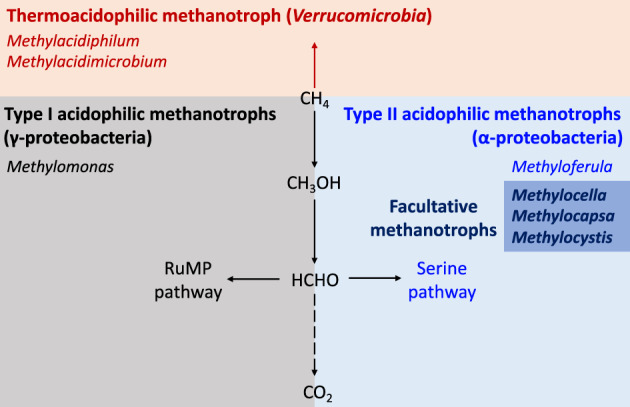
The relationship of acidophilic methanotrophs Types I and II, thermoacidophilic methanotrophs, and facultative methanotrophs. Revised from Figure 1 of Khider et al. (2021). CH_4_, methane; CH_3_OH, methanol; HCHO, formaldehyde; CO_2_, carbon dioxide; RuMP, ribulose monophosphate.

As with neutrophilic methanotrophs, acidophilic methanotrophs contain sMMO and/or pMMO (Belova et al., 2013; Dedysh et al., 2002; Dedysh et al., 2004). For example, *Methylocystis bryophila*, which has an optimal pH between 6.0 and 6.5 (but grows below pH 5.0), contains both pMMO and sMMO, while *Methylocella tundrae* only has sMMO, and effectively grows at pH 5.5–6.0 (Belova et al., 2013; Dedysh et al., 2004). A number of recently discovered species including *Methylocystis bryophila*, *Methylocystis heyeri*, *Methylocapsa aurea*, *Methylocella palustris*, *Metylocella silvestris*, and *Mehtylocella tundrae* are facultative methanotrophs (Nazaries et al., 2013). They can use multi‐carbon compounds such as acetate, organic acids, alcohols, ethane, and propane as carbon and energy sources besides CH_4_ (Farhan Ul Haque et al., 2020). In addition, methanotrophs belonging to the phylum *Verrucomicrobia* have recently been isolated and identified from extremely acidic environments (e.g. below pH 2.0 and/or above a temperature of 50°C; Nazaries et al., 2013).

This review summarizes the occurrence, diversity, and physiology of acidophilic methanotrophs. The occurrence and types of acidophilic methanotrophs are described and their roles in acidic environments are also explored in this review. Recent progress and potential applications of methanotrophs for the biodegradation of chlorinated compounds and other pollutants in acidic groundwater and other low pH environments are also discussed. Scientists are just beginning to understand the implications of facultative growth in acidophilic methanotrophs, and very little research has been done to assess the ability of these organisms to cometabolically degrade CVOCs or other pollutants during growth on secondary substrates, as described later in this review.

## ACIDOPHILIC METHANOTROPHS

### Occurrence

Researchers first began to study acidophilic methanotrophs in natural environments more than 25 years ago. The first evidence of acidophilic methanotrophs was reported by a 16S rRNA‐based study on samples collected from a low pH peat environment (pH 3.6) in 1996 (McDonald et al., 1996). Initially, only a few methanotrophs capable of growth at low pH were isolated in pure culture. This was, in part, due to the use of growth media containing high mineral salt concentrations (1.5–3 g/L) (Dedysh et al., 2002). Peat bogs, where initial enrichment cultures were obtained have not only high acidity but also very low total dissolved solids (TDS) (Dedysh, Panikov, & Tiedje, 1998). Successful isolations of three new acidophilic strains later occurred when low salt medium (containing 50 mg/L mineral salts) was used, and incubation conditions were adjusted to better simulate the oligotrophic and acidic peat bog environment (Dedysh, Panikov, & Tiedje, 1998). Additional strains were subsequently isolated from other acidic environments such as *Sphagnum* peat bogs (Dedysh et al., 2000; Dedysh et al., 2002) and *Sphagnum* tundra peatlands (Dedysh et al., 2004).

Following the aforementioned changes in isolation procedure and growth medium, 10 different pure acidophilic methanotrophs were isolated from other environments including forest soil, forest Cambisol, and collapsed palsa soil (Danilova et al., 2013; Dedysh et al., 2000; Dedysh et al., 2002; Dedysh et al., 2004; Dedysh et al., 2007; Dedysh, Didriksen, et al., 2015; Dunfield et al., 2003; Dunfield et al., 2010; Vorobev et al., 2011). Table 1 summarizes the characteristics of acidophilic methanotrophic isolates that have been reported. These pure strains have also been characterized in terms of their morphology, growth conditions, fatty acid profiles, and enzyme activities. The whole genome sequences of seven of the isolates have been reported (Dedysh, Naumoff, et al., 2015; Esson et al., 2016; Han et al., 2018; Kox et al., 2019; Miroshnikov et al., 2017; Oshkin et al., 2019; Ricke et al., 2005), providing basic information for further study.

**TABLE 1:** Characteristics of isolated acidophilic methanotrophs.

Genus and species	Source	Optimum pH	Optimum temperature range (°C)	MMO expressed	Phenotype	Multi‐carbon substrate	Phylogenetic affiliation	Formaldehyde assimilation	Reference
*Methylocella palustris*	Sphagnum peat bogs	5.0–5.5	15–20	sMMO	Obligate		α‐Proteobacteria	Serine	Dedysh et al. (2000)
*Methylocella siverstris*	Forest cambisol	5.5	15–25	sMMO	Facultative	Organic acids, Alcohols, Ethane, Propane	α‐Proteobacteria	Serine	Dunfield et al. (2003)
*Methylocella tundrae*	Sphagnum tundra peatlands	5.5–6.0	15	sMMO	Facultative	Organic acids, Alcohols	α‐Proteobacteria	Serine	Dedysh et al. (2004)
*Methylocapsa acidiphila*	Sphagnum peat bogs	5.0–5.5	20–24	pMMO	Obligate		α‐Proteobacteria	Serine	Dedysh et al. (2002)
*Methylocapsa aurea*	Forest soil	6.0–6.2	25–30	pMMO	Facultative	Acetate	α‐Proteobacteria	Serine	Dunfield et al. (2010)
*Methylocapsa palsarum*	Collapsed palsa soil	5.2–6.5	18–25	pMMO	Obligate		α‐Proteobacteria	Serine	Dedysh, Didriksen, et al. (2015), Dedysh, Naumoff, et al. (2015)
*Methylocystis heyri*	Sphagnum peat‐bog lake	5.8–6.2	25	pMMO and sMMO	Facultative	Acetate	α‐Proteobacteria	Serine	Dedysh et al. (2007)
*Methylocystis bryophila*	Sphagnum peat‐bog lake	6.0–6.5	25–30	pMMO and sMMO	Facultative	Acetate	α‐Proteobacteria	Serine	Belova et al. (2013)
*Methyloferula stellate*	Sphagnum peat bogs	4.8–5.2	20–23	sMMO	Obligate		α‐Proteobacteria	Serine and RuMP	Vorobev et al. (2011)
*Methylomonas paludis*	Sphagnum peat bogs	5.8–6.4	20–25	pMMO	Obligate		γ‐Proteobacteria	RuMP	Danilova et al. (2013)
*Methylacidiphilum fumariolicum*	Acidic thermal mudpot	0.8–6.0	40–65	pMMO	Facultative	Propane, Ethane	*Verrucomicrobia*		Schmitz et al. (2021)
*Methylacidiphilum infernorum*	Acidic thermal soil	1.0–6.0	40–60	pMMO	Obligate		*Verrucomicrobia*		Schmitz et al. (2021)
*Methylacidiphilum kamchatkense*	Acidic thermal spring	2.0–5.0	37–60	pMMO	Obligate		*Verrucomicrobia*		Islam et al. (2016)
*Methylacidimicrobium cyclopophantes*	Acidic soil	0.6–5.5	44–49	pMMO	Obligate		*Verrucomicrobia*		van Teeseling et al. (2014)
*Methylacidimicrobium fagopyrum*	Acidic soil	0.6–5.5	35–39	pMMO	Obligate		*Verrucomicrobia*		van Teeseling et al. (2014)
*Methylacidimicrobium tartarophylax*	Acidic soil	0.5–5.5	38–43	pMMO	Obligate		*Verrucomicrobia*		van Teeseling et al. (2014)

Abbreviations: pMMO, particulate CH_4_ monooxygenase; sMMO, soluble CH_4_ monooxygenase; RuMP, ribulose monophosphate.

Most acidophilic methanotrophs have been isolated from peat bogs, where CH_4_ is produced through anaerobic decay, and peat moss acidifies its surroundings by taking up calcium and magnesium while releasing hydrogen ions (Danilova et al., 2013; Dedysh et al., 2002). All acidophilic isolates are gram‐negative rods or cocci without flagella or pili. The optimal growth temperature for these methanotrophs is below 30°C, typically around 25°C, making them mesophiles. For example, *Methylomonas*, acidophilic Type 1 organisms, which belong to the γ‐proteobacteria and use the ribulose monophosphate (RuMP) pathway for formaldehyde assimilation, were isolated from an acidic peat bog (Danilova et al., 2013) (Figure 1). Another common methanotroph, *Methylobacter*, which also belongs to γ‐proteobacteria, also have been isolated from acidic environments, such as forest soils below pH 5.0 (Nguyen et al., 2018). This suggests that some genera may adapt to acidic conditions. However, most acidophilic methanotrophs described to date belong to the α‐proteobacteria as Type II methanotrophs, which use the serine pathway for formaldehyde assimilation (Strong et al., 2015). One Type II example, *Methylosinus* was the first identified acid‐tolerant methanotroph from an acidic peat lake (Dedysh, Panikov, & Tiedje, 1998). Several other acidophilic methanotrophs belonging to α‐proteobacteria, including *Methylocella*, *Methylocystis*, *Methylocapsa*, and *Methyloferula*, were discovered in acidic wetlands and peat bogs (Belova et al., 2013; Dedysh et al., 2007). These genera are also known as cold‐tolerant methanotrophs (Dedysh, 2011).

As noted in the introduction, a number of acidophilic methanotrophs have recently been observed to be facultative. Compared to obligate methylotrophs, which only grow on CH_4_ and a limited number of C1 compounds, facultative methanotrophs are known to use not only CH_4_ but also multi‐carbon compounds (i.e. ethane, propane, acetate, ethanol, succinate, and/or organic acids) as sole carbon and energy sources (Dedysh & Dunfield, 2011; Farhan Ul Haque et al., 2020). Facultative methanotrophs primarily belong to α‐proteobacteria including *Methylocystis*, *Methylocella*, *Methylocapsa*, and *Methyloceanibacter* (Belova et al., 2013; Dedysh et al., 2005; Dunfield et al., 2010; Vekeman et al., 2016). *Crenothrix polyspora*, belonging to γ‐proteobacteria, has also been observed to grow on acetate and glucose (Stoecker et al., 2006). Accordingly, facultative methanotrophs might have a competitive advantage over obligate methanotrophs under some conditions due to their metabolic diversity.

Two different genera of methanotrophs (*Methylacidiphilum* and *Methylacidimicrobium*) belonging to the phylum *Verrucomicrobia* have recently been isolated from highly acidic environments (<pH 2.0) (Nazaries et al., 2013; Schmitz et al., 2021; van Teeseling et al., 2014). As shown in Table 1, these methanotrophs can survive under extreme conditions (i.e. at temperatures above 50°C and/or pH below 2.0), and thus are termed ‘thermoacidophilic’. Also, some *Verrucombicrobia* show an ability to grow on propane, ethane, and H_2_ and thus join the expanding group of facultative methanotrophs (Schmitz et al., 2021). One example is *Methylacidiphilum fumariolicum*, which can grow on multi‐carbon compounds including propane and ethane in addition to CH_4_ (Mohammadi et al., 2017; Picone et al., 2020; Pol et al., 2007). These organisms use the Calvin–Benson–Bassham (CBB) cycle to fix carbon dioxide for carbon assimilation unlike Type I and Type II methanotrophs (Op den Camp et al., 2009), and use H_2_ as a growth substrate via hydrogen‐oxidizing enzymes (Mohammadi et al., 2019). The *Verrucomicrobia* phylum has recently been reviewed (Schmitz et al., 2021).

In recent years, the diversity and abundance of acidophilic methanotrophs have been further explored through applications of molecular techniques based on 16S rRNA and/or functional genes (Chen et al., 2008; Ghashghavi et al., 2017). Detection of various novel clones revealed that acidophilic methanotrophs are more widely distributed in the environment than previously thought (Esson et al., 2016; Farhan Ul Haque et al., 2020; Kip et al., 2011; Kip et al., 2012). More discussion of the molecular analysis of acidophilic methanotrophs is provided in ‘Molecular identification’ section and Table 2.

**TABLE 2:** Molecular identification of acidophilic methanotrophs based on presence of PCR‐based assays targeting genes.

Acidophilic methanotrophs	PCR‐based assays targeting genes			
Genus and species	*pmoA*	*mmoX*	*mxaF*	*prmA*
*Methylocella palustris*	/	+	+	/
*Methylocella siverstris*	/	+	+	+
*Methylocella tundrae*	/	+	+	+
*Methylocapsa acidiphila*	+	/	+	/
*Methylocapsa aurea*	+	/	+	/
*Methylocapsa palsarum*	+	/	+	+
*Methylocystis heyri*	+	+	+	/
*Methylocystis bryophila*	+^a^	+	+	/
*Methyloferula stellate*	/	+	+	/
*Methylomonas paludis*	+	/	+	/
*Methylacidiphilum fumariolicum*	+^b^	/	+	/
*Methylacidiphilum infernorum*	+^c^	/	+	/
*Methylacidiphilum kamchatkense*	+^d^	/	+	/
*Methylacidimicrobium cyclopophantes*	+	/	+	+
*Methylacidimicrobium fagopyrum*	+	/	+	/
*Methylacidimicrobium tartarophylax*	+	/	+	+

*Note*: The presence of genes was identified using TBLASTN with representative protein sequences (Gertz et al., 2006) or the Universal Protein Resource (UniProt) database (UniProt Consortium, 2021).

^a^ *pmoA1* and *pmoA2*,

^b^ *pmoA1* and *pmoA3*,

^c^ *pmoA1*, *pmoA2*, and *pmoA3*,

^d^ *pmoA1*, *pmoA2*, *pmoA3*, and *pmoA4*.

### Molecular identification

Fluorescent oligonucleotide probes based on 16S rRNA (Bourne et al., 2000; Dedysh et al., 2003; Kalyuzhnaya et al., 2006), and PCR‐based assays targeting 16S rRNA, *pmoA*, *mmoX*, and methanol dehydrogenase gene, *mxaF* (Chen et al., 2008; Ghashghavi et al., 2017; Hutchens et al., 2004; Kip et al., 2011; Lau et al., 2007; Redmond et al., 2010) are commonly used to identify and investigate the diversity and abundance of acidophilic methanotrophs in different environments. With the PCR‐based assays, many uncultured clones have been detected, demonstrating the wide existence and diversity of acidophilic methanotrophs (Chen et al., 2008; Kip et al., 2011; Knief, 2015; McDonald et al., 1996). The PCR‐based assays targeting genes for acidophilic methanotrophs are shown in Table 2. Recently, the comparative genomic analysis provides insights into the study of acidophilic methanotrophs (Nguyen et al., 2018). Based on reconstructed genomes of uncultured bacteria, one can analyse genomic features including genome sizes, G + C contents, and number of CDSs, and then compare to the reference genomes to identify the uncultured acidophilic methanotrophs in a given environmental sample (Nguyen et al., 2018).

### Characteristics of pure strains

**FIGURE 2:**
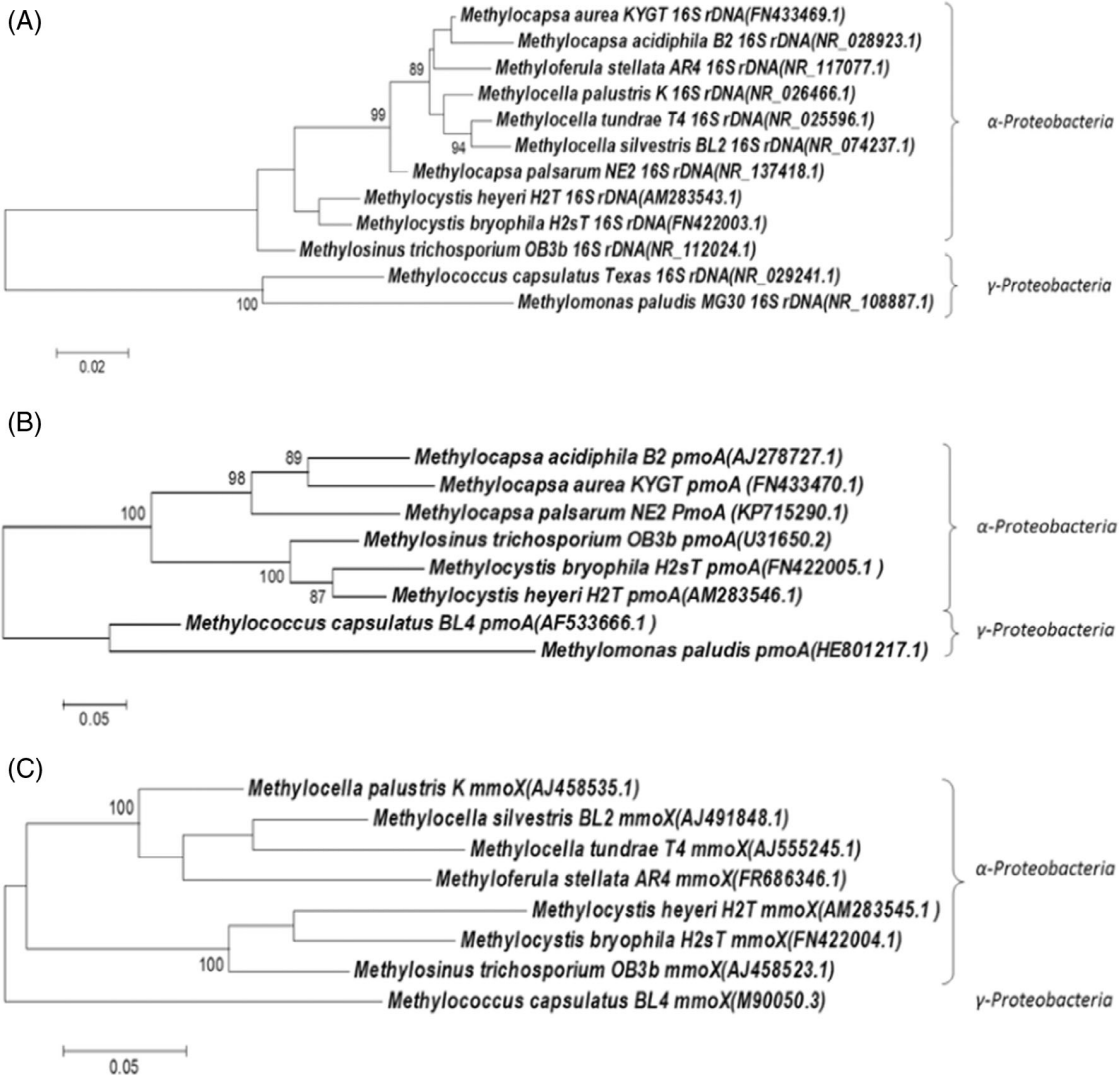
Phylogenetic tree based on (A) 16S rDNA, (B) *pmoA*, and (C) *mmoX*.

Several pure strains of acidophilic methanotrophs have been isolated as summarized in Table 1. Adapting the molecular identification approach in ‘Molecular identification’, a comparative sequence analysis based on 16S rDNA, *pmoA*, and *mmoX* of the isolated acidophilic methanotrophs in Table 1 was performed (Figure 2). Each phylogenetic tree was generated with 16S rDNA, *pmoA*, and *mmoX* gene sequences of 10 strains in GenBank using the neighbour‐joining software MEGA. The evolutionary relationship among these 10 strains is provided in Figure 2A. The analysis shows that species belonging to class α‐proteobacteria have a relatively high similarity (73%–87%) in the *pmoA* gene (Figure 2B) as well as the *mmoX* gene (80%–86% similarity) (Figure 2C).

As previously described, three acidophilic methanotrophs were successfully isolated from peat bog environments in 1998 using a medium with low ionic strength (Dedysh, Panikov, Liesack, et al., 1998). These strains all belonged to the genus *Methylocella*, with *Methylocella palustris* being the first species identified in this genus (Dedysh et al., 2000). *Methylocella palustris* was found to only express sMMO because no products were observed during PCR with pMMO‐targeted primers; hybridization with a *pmoA* probe also was negative. This observation ran counter to the previous belief that all methanotrophs contained the *pmoA* gene and expressed pMMO (Dedysh et al., 2000). Analysis of their 16S rDNA sequences revealed that these strains may have evolved from the same ancestors, the acidophilic heterotrophic bacteria *Beijerinckia indica* and *Rhodopseudomonas acidophila* (Dedysh, Panikov, Liesack, et al., 1998). Also, based on molecular analysis of *mmo*X genes of the *Methylocella* strains, they appear more closely related to *Methyloferula* spp. than to the known *Methylosinus‐Methylocystis* cluster in the α‐proteobacteria (Figure 2C). The analysis showed that the genus of *Methylosinus* and *Methylocystis* are more closely related to each other than to *Methylocella*.

In 2002, another species belonging to a novel genus, *Methylocapsa acidiphila* B2^T^, was isolated from an acidic *Sphagnum* peat bog (Dedysh et al., 2002). This bacterium belongs to the α‐proteobacteria and has 97.3% similarity to the 16S rRNA of *Methylocella palustris* K^T^ (Figure 2A). However, there was only 7% DNA–DNA hybridization between *Methylocapsa acidiphila* B2^T^ and *Methylocella palustris* K^T^. *Methylocapsa acidiphila* B2^T^ only expressed pMMO, which was different from *Methylocella palustris* K^T^ which only expressed sMMO (Dedysh et al., 2002). *Methylocapsa aurea* KYG^T^, another acidophilic methanotroph in this genus, was observed to be facultative, capable of using acetate as a carbon source in addition to CH_4_ (Dunfield et al., 2010; Farhan Ul Haque et al., 2020). This capability distinguishes the strain from closely related *Methylocapsa acidiphila* B2^T^ which is unable to grow on non‐C1 substrates. *Methylocapsa aurea* KYG^T^ also proved to be more sensitive to pH and salt concentration than other strains in the genus, with optimum pH in a narrow range of 6.0–6.2 and not surviving at pH <5 (Dunfield et al., 2010).

*Methylocella silvestris*, which was isolated from an acidic forest Cambisol, is morphologically and phenotypically similar to *Methylocella palustris* K^T^ (Dunfield et al., 2003). This organism is a facultative methanotroph that possesses only a form of sMMO that is produced by the *mmoX* gene. As with many methanotrophs that have both sMMO and pMMO, the expression of sMMO is affected by the concentration of copper in the growth medium. However, in this bacterium, the expression of sMMO was not downregulated by copper (Theisen et al., 2005). *Methylocella silvestris* was the first methanotroph observed to be capable of using propane as a carbon source while constitutively expressing sMMO (Crombie & Murrell, 2014), although other works showed that the expression of *mmoX* was repressed during growth on acetate (Rahman et al., 2011). During propane oxidation in this strain, sMMO and propane monooxygenase (PrMO) are both expressed (Dunfield & Dedysh, 2014). The substrates that can be utilized by *Methylocella silvestris* have been expanded to include 2‐propanol, 1,2‐propanediol, acetone, methyl‐acetate, acetol, glycerol, propionate, tetrahydrofuran, and gluconate, as well as the gaseous alkanes ethane and propane (Dunfield & Dedysh, 2014).

Another methanotroph capable of growth at low pH, *Methylocella tundrae*, uses sMMO to grow on CH_4_ (Dedysh et al., 2004), but genome analysis indicates that this bacterium also carries a PrMO gene cluster in its megaplasmids (Kox et al., 2019). While two species of *Methylocella* possess PrMO allowing them to grow on multi‐carbon substrates, the PrMO gene cluster of *Methylocella silvestris* is in its genome while that of *Methylocella tundrae* is in its megaplasmids. A comparative genomic study of *Methylocella* indicates a close relationship to *Beijerinckia indica*, which are acidophilic nitrogen‐fixing bacteria but non‐methanotrophs (Tamas et al., 2014). *Methylocella* and *Beijerinckia* have been suggested to have evolved from a common obligate methanotroph, but each has expanded beyond just CH_4_ as a substrate; in the case of *Beijerinckia indica*, losing CH_4_ metabolism altogether (Dunfield & Dedysh, 2014; Tamas et al., 2014).

Species in the genus *Methyloferula* are thought to only possess sMMO (Vorobev et al., 2011). Unlike *Methylocella silvestris*, which contains an additional soluble diiron monooxygenase for propane oxidation, *Methyloferula stellata* is an obligate methanotroph (Farhan Ul Haque et al., 2020; Vorobev et al., 2011). However, the 16S rRNA gene and sMMO sequence analysis the between two genera suggested that they are closely related (Figure 2A,C).

*Methylocystis* possess both pMMO and sMMO, and the expression of these MMOs is affected by the concentration of copper as typical of many methanotrophs (Table 2). *Methylocystis heyri* and *Methylocystis bryophila* are two moderately acidophilic methanotrophs in this genus (Belova et al., 2013; Dedysh et al., 2007). Interestingly, *M. heyri* shows a unique profile of phospholipid fatty acids, containing both 18:1ω8c (i.e. a common type of α‐proteobacterial methanotrophs) and 16:1ω8c (i.e. a common type of γ‐proteobacterial methanotrophs) (Dedysh, 2011). These two species are also facultative methanotrophs, and they can express different forms of MMOs, perhaps explaining why they are widespread in the environment (Han et al., 2018; Leng et al., 2015). As an example, *M. bryophila* contains two different *pmoA* genes, *pmoA1*, and *pmoA2*, responsible for pMMO1 and pMMO2, respectively. The pMMO2 shows a higher CH_4_ affinity than pMMO1, potentially allowing growth over a wide range of CH_4_ concentrations (Baani & Liesack, 2008).

*Methylomonas paludis* was the first acidophilic methanotroph discovered that belongs to the γ‐proteobacteria (Danilova et al., 2013). The absence of motility and the ability to grow under acid conditions makes it different from other species in *Methylomonas* (Danilova et al., 2013). Figure 2A,B shows the long distance between *Methylomonas paludis* and other acidophilic methanotrophs based on 16S rDNA and *pmoA* genes, respectively. Based on 16S rRNA sequences, *Methylomonas paludis* has 80%–90%, homology to the acidophilic methanotrophic species in class α‐proteobacteria (Figure 2A). The *pmoA* gene sequence of *Methylomonas paludis* shows 71% homology to that of *Methylococcus capsulatus*; both species belong to the class of γ‐proteobacteria (Figure 2B). Recently, two novel acid‐tolerant moderately thermophilic methanotrophs, *Methylococcaceae* strain BFH1 and BFH2, belonging to γ‐proteobacteria have been isolated from tropical soils with CH_4_ leakage (Islam et al., 2016).

### Mechanisms of survival at low pH

The mechanisms for the survival of acidophilic methanotrophs, particularly those capable of growth under extremely acidic conditions (e.g. pH < 3) are not well understood. However, the general literature on acidophilic organisms indicates a number of different potential strategies that allow existence under highly acidic conditions, including reversed membrane potentials, extremely impermeable membranes, and the occurrence of numerous secondary transporters (Baker‐Austin & Dopson, 2007). The majority of these hypotheses are derived from genome and biochemical analyses. The composition of fatty acids and lipids in the cell membrane of acidophiles has been observed to differ from more neutrophilic organisms supporting the critical nature of membrane structure in low pH survival (Sharma et al., 2016). Other proposed, but as yet unproven, mechanisms to maintain neutral cytoplasmic pH include buffering and sequestration of protons inside the cytoplasm (Sharma et al., 2016). Future studies are needed to specifically investigate mechanisms of pH tolerance in acidophilic methanotrophs and to compare these approaches to those of more widely studied acidophiles.

### Heavy metal resistance of acidophilic methanotrophs

Acidophilic microbes often have enhanced heavy metal resistance due to the likelihood of encountering high concentrations of many metals at low pH based on solubility considerations (Nordstrom et al., 2000). Some acidophiles have developed efflux pumping systems for heavy metals and/or expressed heavy metal resistance or reductase genes (Dopson & Holmes, 2014; Mangold et al., 2013). Heavy metal resistance genes include *copCD*, *terB*, and *merR* which are responsible for copper, tellurite, and mercury resistance, respectively. *Methylobacter* sp. also has genes for specific reductases including *arsC* (arsenate reductase). Thus, this strain has multiple strategies to protect against the toxicity of heavy metals.

## METHANOTROPHIC ACTIVITY IN ACIDIC WETLANDS

Acidic peat bogs, which are dominated by the mosses of the genus *Sphagnum*, are one of the most extensive types of wetlands (Dedysh, 2009; Kolb & Horn, 2012). These bogs, mostly located from 50°N to 70°N latitude, are a significant source of CH_4_, emitting 100–237 Gt per year (Dedysh, 2009; Kolb & Horn, 2012). Type II acidophilic methanotrophs, including *Methylocystis*, *Methylocapsa*, and *Methylocella*, have been most commonly identified in the acidic wetlands and are likely key contributors to the regulation of CH_4_ fluxes from these environments, reducing the overall impact on climate change (Dedysh, 2009). Some methanotrophs in acidic bogs are thought to have a symbiotic relationship with *Sphagnum*, but the interactions between the mosses and methanotrophs are not completely understood (Dedysh, 2011). *Methylocystis*, one of the active and dominant methanotrophs in acidic wetlands, has been highlighted as a facultative methanotroph, showing the ability to use acetate for growth in addition to CH_4_ (Kolb & Horn, 2012). The strain can change its growth substrate to acetate from CH_4_ during periods of CH_4_ depletion in acidic environments (Dedysh, 2009, 2011). Interestingly, *Methylocystis* contains a distinct *pmoA2* gene, which differs from the *pmoA* gene, by encoding a pMMO capable of oxidizing CH_4_ at very low environmental concentrations (Baani & Liesack, 2008). Due to the high CH_4_ affinity of *pmoA2*, species containing this gene/enzyme are likely to consume CH_4_ at atmospheric concentrations (Kolb & Horn, 2012). Thus, this organism appears capable of survival under low and CH_4_‐limiting conditions and can switch to an alternate substrate if CH_4_ is absent. Type I methanotrophs belonging to γ‐proteobacteria also have been identified in acidic wetlands even though these environments are commonly dominated by the Type II methanotrophs as just described (Dedysh, 2009; Kolb & Horn, 2012; Nguyen et al., 2018). *Verrucomicrobial* methanotrophs, which have been isolated from extremely acidic conditions, are also found in acidic wetlands, but the overall distribution of these methanotrophs is still unknown (Dedysh, 2009). Further studies are required to better understand the diversity, distribution, and activity of methanotrophs in acidic wetlands.

## GROUNDWATER POLLUTION AND METHANOTROPHS

### Biodegradation of groundwater contaminants by MMO

Cometabolic biodegradation typically occurs when one or more broad‐specificity enzymes (typically monooxygenases) are induced in bacteria—enzymes that allow such bacteria to grow on a primary substrate (e.g. CH_4_, ethane, propane, and butane), yet also to biodegrade a range of other non‐growth compounds, including many contaminants of concern (Alexander, 1994). Numerous different organisms are capable of cometabolic biodegradation including species of *Pseudomonas*, *Burkholderia*, and *Rhodococcus*, but methanotrophs have perhaps received the most study in this regard (Chu & Alvarez‐Cohen, 1998; Halsey et al., 2005; Mahendra & Alvarez‐Cohen, 2006; Singh & Singh, 2017; Wang & Chu, 2017).

**FIGURE 3:**
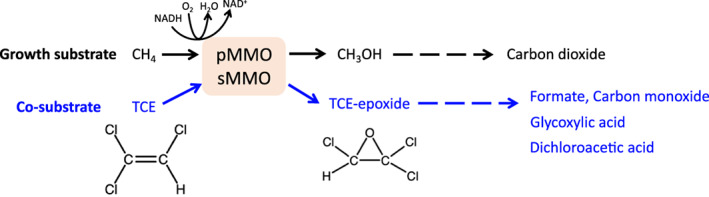
Cometabolic degradation pathway of trichloroethylene (TCE). CH_4_, methane; CH_3_OH, methanol; pMMO, particulate methane monooxygenase; sMMO, soluble methane monooxygenase.

In the 1980s, researchers found that CH_4_ stimulated TCE degradation in aerobic sediment columns and in a mixed methanotrophic culture (Fogel et al., 1986; Wilson & Wilson, 1985). These studies were important because, at the time, chlorinated solvents such as TCE were perhaps the most important and widespread emerging environmental contaminants, and little was known concerning their biodegradation. In 1988, two pure methanotrophs capable of cometabolically biodegrading TCE were isolated from groundwater samples (Little et al., 1988). The two common monooxygenases possessed by methanotrophs (pMMO and sMMO) each were observed to catalyse the biodegradation of TCE, although rates were found to be appreciably more rapid via sMMO (Lee et al., 2006). During TCE oxidation by either sMMO or pMMO, the initial step is oxidation to TCE‐epoxide, followed by spontaneous and or further enzymatic degradation of the epoxide to multiple products including formate, carbon monoxide, glyoxylic acid, and dichloroacetic acid (Figure 3).

MMOs (pMMO and sMMO) have been observed to biodegrade a variety of other groundwater contaminants besides TCE, including many different chlorinated aliphatics (e.g. VC, *cis*‐DCE, 1,2,3‐trichloropropane [TCP], and chloroform [CF]), aromatics such as BTEX, some aromatic hydrocarbons, and numerous other organic pollutants (Bosma & Janssen, 1998; Hand et al., 2015; Im & Semrau, 2011; Malachowsky et al., 1994; Wackett et al., 1989; Wang & Chu, 2017). *cis*‐1,2‐DCE and VC are degradation intermediates that occur commonly during anaerobic biodegradation of TCE (Schäfer et al., 2003; Semprini et al., 1990; Wüst et al., 1999) (Table 3). TCP was used as a chemical intermediate in organic synthesis, as a solvent, and as an extractive agent. It has been detected in hundreds of surface water and drinking water sources in the US, at levels of 0.1–100 μg/L. Typically, remediation of TCP‐contaminated sites is difficult due to its physiochemical properties (Salter et al., 2010).

**TABLE 3:** Representative compounds degraded by monooxygenases.

	pMMO		sMMO		PrMO		Reference
Chlorinated aliphatic hydrocarbons							
TCE	+	*Methylosinus trichosporium* OB3b *Methylocystis* strain SB2	+	*Methylosinus trichosporium* OB3b *Methylocella* spp.^a^ *Methylocella palustris* ^a^	+	*Mycobacterium vaccae* JOB5 *Rhodococcus jostii* RHA1 *Rhodococcus rhodochrous*	Anderson and McCarty (1997), Hatzinger et al. (2017), Im and Semrau (2011), Malachowsky et al. (1994), Oldenhuis et al. (1991), Shao et al. (2019), Tsien et al. (1989), Wackett et al. (1989)
TCP	N/A		+	*Methylosinus trichosporium* OB3b	+	*Rhodococcus jostii* RHA1 *Rhodococcus rubber* ENV425 *Mycobacterium vaccae* JOB5 *Sphingopyxis* sp. AXE‐A	Bosma and Janssen (1998), Wang and Chu (2017)
VC	+	*Methylosinus trichosporium* OB3b *Methylocystis* strain SB2 *Methylomonas* sp. Strain JS1^a^	+	*Methylosinus trichosporium* OB3b *Methylocystis* sp. Strain MJC1^a^	+	*Mycobacterium vaccae* JOB5 *Rhodococcus rhodochrous*	Anderson and McCarty (1997), Choi et al. (2021), Hand et al. (2015), Im and Semrau (2011), Malachowsky et al. (1994), Tsien et al. (1989), Wackett et al. (1989)
Chloroform	+	*Methylocystis* strain SB2	+	*Methylosinus trichosporium* OB3b	+	*Rhodococcus rhodochrous*	Im and Semrau (2011), Malachowsky et al. (1994), Oldenhuis et al. (1989)
Aromatic hydrocarbons							
Naphthalene	/	*Methylosinus trichosporium* OB3b	+	*Methylosinus trichosporium* OB3b	N/A		Brusseau et al. (1990)
BTEX	+	*Methylocystis* sp. *Methylosinus trichosporium* OB3b	+	*Methylococcus capsulatus* *Methylosinus trichosporium* OB3b	+	*Rhodococcus erythropolis*	Hesselsoe et al. (2005), Lee et al. (2011), Kulikova and Bezborodov (2000), Wilkins et al. (1994)
Other compounds							
1,4‐Dioxane	/	*Methylosinus trichosporium* OB3b	+ /	*Methylosinus trichosporium* OB3b	+	*Mycobacterium vaccae* JOB5 *Rhodococcus jostii* RHA1	Hand et al. (2015), Mahendra and Alvarez‐Cohen (2006)
MTBE	N/A		+	CH4 grown microbial consortia	+	*Rhodococcus* sp. Strain ENV425	Hesselsoe et al. (2005), Steffan et al. (1997)
NDMA	/	*Methylosinus trichosporium* OB3b	+	*Methylosinus trichosporium* OB3b	+	*Mycobacterium vaccae* JOB5	Sharp et al. (2005)
‐	‐		‐		‐		
Pesticides	+	CH4‐oxidizing cultures	+	CH4‐oxidizing cultures	N/A		Hedegaard et al. (2020)
Pharmaceuticals	+	*Methylocystis parvus*	+	CH4‐oxidizing cultures	N/A		Benner et al. (2015)

Abbreviations: BTEX, Benzene, toluene, ethylbenzene and xylene; ‐; MTBE, methyl tertiary‐butyl ether; NDMA, *N*‐nitroso‐dimethylamine; pMMO, particulate CH_4_ monooxygenase; PrMO, propane monooxygenase; sMMO, soluble CH_4_ monooxygenase; TCE, trichloroethylene; TCP, trichloropropane; VC, vinyl chloride.

^a^ Acidophilic methanotrophs.

^b^ Able to degrade; +, Not able to degrade /; N/A, no related literature. Results in the literature differ for 1,4‐dioxane and sMMO, thus the + / designation.

The rates and extents of pollutant degradation by methanotrophs vary based on the MMO expressed. sMMO‐expressing methanotrophs typically degrade more compounds than pMMO‐expressing cells and degrade such compounds at faster initial rates (Alvarez‐Cohen & McCarty, 1991; Anderson & McCarty, 1997; Lee et al., 2006; Lontoh & Semrau, 1998; Shukla et al., 2009; Tsien et al., 1989). Thus, much of the early literature focused on the utility of sMMO‐expressing organisms for pollutant degradation (Alvarez‐Cohen & McCarty, 1991; Lee et al., 2006; McDonald et al., 1997; Shigematsu et al., 1999). For example, sMMO but not pMMO can oxidize naphthalene (Brusseau et al., 1990; Chang et al., 2002). sMMO is also able to oxidize several emerging contaminants, including methyl tertiary‐butyl ether (MTBE) (Hesselsoe et al., 2005; Steffan et al., 1997) and *N*‐nitrosodimethylamine (NDMA) among others (Sharp et al., 2005). Pharmaceuticals such as sulfamethoxazole (Benner et al., 2015) and ibuprofen (Dawas‐Massalha et al., 2014), as well as a variety of pesticides including bentazone, dichlorprop, and chlorophenoxy herbicide (Hedegaard et al., 2020; Papadopoulou et al., 2019), are also degraded by sMMO. 1,4‐Dioxane, which is commonly used as a stabilizer for chlorinated solvents such as 1,1,1‐trichloroethane (TCA), was originally reported to be oxidized by sMMO (Mahendra & Alvarez‐Cohen, 2006). Later studies with pure cultures and pure sMMO enzyme revealed, however, that 1,4‐dioxane is not a substrate for sMMO and is unlikely to be degraded by methanotrophs in the environment (Hatzinger et al., 2017).

Other studies suggest that pMMO‐expressing methanotrophs survive more readily in environments with complex mixtures and/or high concentrations of pollutants such as chlorinated ethenes (Semrau, 2011). It is thought that sMMO‐expressing methanotrophs accumulate toxic products resulting from pollutant oxidation (e.g. epoxides from chlorinated ethenes) faster than pMMO‐expressing cells, and thus may experience higher rates of cell toxicity due to epoxide‐mediated damage to the sMMO enzyme and other cellular macromolecules (Chu & Alvarez‐Cohen, 1999; Fox et al., 1990; Semrau, 2011). In this instance, the overall pollutant degradation by sMMO‐expressing methanotrophs may be less than observed for those expressing only pMMO (Lee et al., 2006). Due to the potential for cell toxicity for both sMMO and pMMO from chlorinated ethenes, cometabolic treatment of these compounds in groundwater is likely to be more effective when they are present at relatively low concentrations (i.e. tens to hundreds of μg/L). It should also be noted, however, that the toxicity of the relevant epoxides from chlorinated ethenes vary widely by compound.

### Contamination and remediation in acidic groundwater

Most of the aforementioned studies of pollutant degradation by methanotrophs have been conducted under neutral pH conditions. There is comparatively little information on the capabilities of acidophilic methanotrophs to biodegrade pollutants, such as TCE or others in groundwater. This is particularly important because low pH groundwater is common throughout the Northern Atlantic Coastal Plain aquifer system in the United States, which occurs from Long Island, New York through most of North Carolina (Denver et al., 2014). Of 419 groundwater samples collected by USGS from this aquifer system, 250 (60%) were reported to have pH values of 5.5 or below (data archive; https://pubs.usgs.gov/circ/1353/). This aquifer system, which includes a number of large military facilities and some large urban areas, is also significantly impacted by CVOCs (Denver et al., 2014). A second aquifer system composed of similarly semi‐consolidated sands with poor buffering capacity is the Gulf of Mexico Coastal Plain aquifer system, running from Georgia, through the panhandle of Florida, and to the southern tip of Texas (DeSimone et al., 2014). This system also has many sites with CVOCs in low‐pH groundwater and/or groundwater with very poor buffering capacity. Many other locations in the United States also have locally acidic groundwater.

As noted in the Introduction, *in situ* bioremediation of CVOC‐contaminated sites is often performed by adding high concentrations of carbon sources (such as lactate or emulsified vegetable oils) to stimulate natural or introduced *Dehalococcoides* spp., to anaerobically degrade PCE and TCE to ethene (Chen et al., 2022; Stroo & Ward, 2010; Yang et al., 2017). However, one significant issue with anaerobic bioremediation of CVOCs is that complete reductive dechlorination (i.e. PCE or TCE to ethene) by *Dehalococcoides* spp. is typically inhibited at pH < ~5.5 (Eaddy, 2008; Lacroix et al., 2014; Rowlands, 2004; Steffan & Vainberg, 2013; Vainberg et al., 2009; Yang, 2012; Yang et al., 2017). Most organisms or consortia capable of reducing TCE have pH optima between ~6.5 and 8 and do not effectively dehalogenate this compound, or other chlorinated ethenes to ethene below pH 5.5. Supporting organisms, such as those that produce cobalamin required by *Dehalococcoides* spp., also may be inhibited at low pH (Puentes Jácome et al., 2019). In a study by Yang (2012), only one strain was observed to effectively dehalogenate PCE at pH 5.5 (*Sulfospirillum multivorans*), and this organism degraded PCE to *cis*‐DCE as a terminal product. VC, a known carcinogen with a U.S. Federal Maximum Contaminant Level (MCL) of 2 μg/L, is also a potential terminal product of a stalled anaerobic biodegradation of chlorinated ethenes in acidic environments. As a result, anaerobic bioremediation is largely ineffective at reducing chlorinated ethenes to ethene in naturally acidic aquifers. In addition, when carbon sources are added to groundwater aquifers in large quantities to promote reductive dechlorination, the formation of organic and inorganic acids can cause pH in poorly buffered aquifers to fall below optimal levels, resulting in incomplete or stalled dechlorination (McCarty et al., 2007).

Aquifer buffering has been attempted in some instances to increase groundwater pH for remediation purposes (Hatzinger et al., 2006; Schaefer et al., 2010), but the amount of buffer required makes this process cost prohibitive for other than small sites. In some instances, a strong base (e.g. NaOH) has been used to increase aquifer pH, but this can easily result in overshooting the desired pH range, and subsequently causing significant precipitation reactions as well as dissolution of natural organics. Because of the difficulty in applying typical in situ anaerobic bioremediation technologies in acidic groundwater, other remediation strategies are required to treat CVOCs in these aquifers. The application of methane with or without exogenous acidophilic methanotrophs may represent an appropriate strategy in many such environments as described below. It is also possible that these organisms are already contributing to the natural attenuation of CVOCs in acidic groundwater, but this process is largely unrecognized.

## POTENTIAL BIOREMEDIATION APPLICATIONS IN LOW pH AQUIFERS

The potential for acidophilic methanotrophs to biodegrade pollutants is largely unknown, and evaluation of potential applications of these organisms has just begun in recent years (Choi et al., 2021; Semrau, 2011; Shao et al., 2019; Szwast, 2021). This pursuit is important, particularly for chlorinated solvents because, as previously noted, traditional bioremediation approaches are not as effective at low pH for completely dechlorinating CVOCs, particularly chlorinated ethenes. Secondly, the recent observation that a number of methanotrophs, including six different acidophilic strains, are facultative (Farhan Ul Haque et al., 2020) enhances the potential for natural biodegradation of pollutants (e.g. in environments without CH_4_ but with alternate substrates), as well as new ways to enhance pollutant bioremediation in acidic environments. The critical questions are (1) are acidophilic methanotrophs present in the contaminated groundwater environments (2) do they possess forms of MMO capable of biodegrading TCE and other contaminants after growth on CH_4_; and (3) are these MMO(s) expressed and active using alternate substrates. One methanotroph, *Methylocystis* strain SB2, which was isolated from a neutral medium, constitutively expressed pMMO growing with ethanol and successfully degraded VC, *trans*‐dichloroethylene (*t*‐DCE), TCE, and 1,1,1‐trichloroethane (1,1,1‐TCA) through cometabolism (Im & Semrau, 2011; Jagadevan & Semrau, 2013).

However, studies on the cometabolism of pollutants by methanotrophs in acidic environments are lacking (Table 3). One initial study by our group demonstrated that *Methylocella palustris* degraded TCE and several other halogenated organics including 1,2‐dibromoethane (EDB), chloroform, VC, and *cis*‐DCE, but not perchloroethene (PCE) or 1,4‐dioxane at pH 5.0 (Hatzinger et al., 2017). Further work showed that methanotrophs capable of degrading TCE existed in multiple acidic aquifers (Shao et al., 2019). Using stable‐isotope‐probing techniques, phylogenetically diverse active methanotrophs were detected in low‐pH aquifer microcosms (Shao et al., 2019). The methanotrophs in these microcosms included *Methylomonas*, *Methylocaldum*, *Methylobacter*, *Methylosinus*, and *Methylococcus*, which belong to γ‐proteobacteria or α‐proteobacteria, but are not necessarily related to other known acidophilic methanotrophs. It is likely that one or more of these organisms facilitated the observed cometabolic biodegradation of TCE. In addition, a recent study showed the cometabolic biodegradation of VC in acidic environments by isolated acidophilic methanotrophs from acidic peat soils (Choi et al., 2021).

The quantification of natural attenuation of TCE under oxidative conditions has been challenging due to the general absence of easily detected daughter products akin to the production of *cis*‐DCE, VC, and ethene via reductive dehalogenation. However, a method developed at Clemson University that utilizes ultrapure ^14^C‐TCE to quantify the oxidative conversion of this CVOC to ^14^CO_2_ and soluble ^14^C‐labelled daughter products, has overcome this limitation (Mills IV et al., 2018). This technique has recently been applied to estimate TCE degradation rates under aerobic conditions in an acidic aquifer in Maryland with pH ranging from 4.3 to 6.1 (Szwast, 2021). Biostimulation via CH_4_ and inorganic nutrient addition also was assessed. First‐order rate constants ranging from 0.012 to 3.0 year^−1^ were calculated (half‐lives of 0.23–59 years) across the range of microcosms from three locations. The highest rate constants were generally in treatments with CH_4_, and nutrients added, but TCE degradation was also observed in treatments representative of in situ conditions (i.e. no additions). These data support the hypothesis that methanotrophs are important but largely unrecognized contributors to aerobic cometabolism of TCE under low pH conditions.

## CONCLUSIONS AND FUTURE WORK

This review focuses on the distribution, diversity, and potential bioremediation activities of acidophilic methanotrophs. Since acidophilic methanotrophs were first discovered more than 25 years ago, great progress has been made in describing their characteristics and distribution in low pH environments, including peat bogs, wetlands and lakes, thermal soils, and springs, and more recently, groundwater aquifers. New genera have been discovered with unique physiological characteristics, including the potential for utilizing longer‐chain compounds for substrates. Compared to neutral pH environments, however, there is a relative dearth of information concerning the potential for methanotrophs to biodegrade CVOCs and other pollutants under acidic conditions either naturally or via biostimulation or bioaugmentation. This is particularly important for TCE and many other CVOCs because reductive biodegradation processes are not particularly effective below pH 5.5.

There are a number of important areas that require further research. These include the isolation and identification of pure methanotrophic cultures from low pH groundwater environments, as to date no such organisms are available for study. This is critically important for understanding the fundamental physiological and biodegradative capabilities of methanotrophs in groundwater environments. Another critical area is the assessment of facultative growth of acidophilic methanotrophs in groundwater and whether alternate (i.e. non‐CH_4_) substrates can be utilized as carbon sources to promote methanotrophic degradation of TCE and other CVOCs. This question is critical to our understanding of the natural attenuation of these pollutants in aerobic aquifers, a largely unstudied area. Further studies on community diversity and dynamics of methanotrophs and associated organisms (i.e. non‐methanotrophs) that may contribute to pollutant biodegradation are also of interest. Finally, as we come to better understand the potential of these organisms for degrading persistent pollutants, field studies of biostimulation (e.g. CH_4_, nutrient, and oxygen addition) for enhanced pollutant remediation in acidic aquifers are required so that this approach can ultimately be optimized and utilized for large‐scale treatment of CVOCs and other pollutants, much the way reductive dehalogenation has been widely applied for CVOC treatment in neutral pH environments.

## CONFLICT OF INTEREST STATEMENT

The authors declare no conflicts of interest.

## References

[emi413156-bib-0001] Alexander, M. (1994) Cometabolism. In: Biodegradation and Bioremediation. New York: Academic Press.

[emi413156-bib-0002] Alvarez‐Cohen, L. & McCarty, P. (1991) Effects of toxicity, aeration, and reductant supply on trichloroethylene transformation by a mixed methanotrophic culture. Applied and Environmental Microbiology, 57, 228–235. 2036009 10.1128/aem.57.1.228-235.1991 PMC182690

[emi413156-bib-0003] Anderson, J.E. & McCarty, P.L. (1997) Transformation yields of chlorinated ethenes by a methanotrophic mixed culture expressing particulate methane monooxygenase. Applied and Environmental Microbiology, 63, 687–693. 9023946 10.1128/aem.63.2.687-693.1997 PMC168358

[emi413156-bib-0004] Baani, M. & Liesack, W. (2008) Two isozymes of particulate methane monooxygenase with different methane oxidation kinetics are found in *Methylocystis* sp. strain SC2. Proceedings of the National Academy of Sciences of the United States of America, 105, 10203–10208. 18632585 10.1073/pnas.0702643105 PMC2481331

[emi413156-bib-0005] Baker‐Austin, C. & Dopson, M. (2007) Life in acid: pH homeostasis in acidophiles. Trends in Microbiology, 15, 165–171. 17331729 10.1016/j.tim.2007.02.005

[emi413156-bib-0006] Belova, S.E. , Kulichevskaya, I.S. , Bodelier, P.L. & Dedysh, S.N. (2013) *Methylocystis bryophila* sp. nov., a facultatively methanotrophic bacterium from acidic Sphagnum peat, and emended description of the genus *Methylocystis* (ex Whittenbury et al. 1970; Bowman et al. 1993). Journal of Systematics and Evolution, 63, 1096–1104. 10.1099/ijs.0.043505-0 22707532

[emi413156-bib-0007] Benner, J. , De Smet, D. , Ho, A. , Kerckhof, F.‐M. , Vanhaecke, L. , Heylen, K. et al. (2015) Exploring methane‐oxidizing communities for the co‐metabolic degradation of organic micropollutants. Applied Microbiology and Biotechnology, 99, 3609–3618. 25487887 10.1007/s00253-014-6226-1

[emi413156-bib-0008] Bosma, T. & Janssen, D.B. (1998) Conversion of chlorinated propanes by *Methylosinus trichosporium OB3b* expressing soluble methane monooxygenase. Applied Microbiology and Biotechnology, 50, 105–112. 10.1128/aem.55.11.2819-2826.1989 PMC203175 2624462

[emi413156-bib-0009] Bourne, D.G. , Holmes, A.J. , Iversen, N. & Murrell, J.C. (2000) Fluorescent oligonucleotide rDNA probes for speci¢c detection of methane oxidising bacteria. FEMS Microbiology Ecology, 31, 29–38. 10620716 10.1111/j.1574-6941.2000.tb00668.x

[emi413156-bib-0010] Brusseau, G.A. , Tsien, H.‐C. , Hanson, R.S. & Wackett, L.P. (1990) Optimization of trichloroethylene oxidation by methanotrophs and the use of a colorimetric assay to detect soluble methane monooxygenase activity. Biodegradation, 1, 19–29. 1368139 10.1007/BF00117048

[emi413156-bib-0011] Cai, Y. , Zheng, Y. , Bodelier, P.L. , Conrad, R. & Jia, Z. (2016) Conventional methanotrophs are responsible for atmospheric methane oxidation in paddy soils. Nature Communications, 7, 11728. 10.1038/ncomms11728 PMC4895445 27248847

[emi413156-bib-0012] Chang, S.W. , Hyman, M.R. & Williamson, K.J. (2002) Cooxidation of naphthalene and other polycyclic aromatic hydrocarbons by the nitrifying bacterium, *Nitrosomonas europaea* . Biodegradation, 13, 373–381. 12713129 10.1023/a:1022811430030

[emi413156-bib-0013] Chen, G. , Kara Murdoch, F. , Xie, Y. , Murdoch, R.W. , Cui, Y. , Yang, Y. et al. (2022) Dehalogenation of chlorinated ethenes to ethene by a novel Isolate, ‘*Candidatus* Dehalogenimonas etheniformans’. Applied and Environmental Microbiology, 88, e00443–e00422. 35674428 10.1128/aem.00443-22 PMC9238427

[emi413156-bib-0014] Chen, Y. , Dumont, M.G. , Neufeld, J.D. , Bodrossy, L. , Stralis‐Pavese, N. , McNamara, N.P. et al. (2008) Revealing the uncultivated majority: Combining DNA stable‐isotope probing, multiple displacement amplification and metagenomic analyses of uncultivated *Methylocystis* in acidic peatlands. Environmental Microbiology, 10, 2609–2622. 18631364 10.1111/j.1462-2920.2008.01683.x

[emi413156-bib-0015] Choi, M. , Yun, T. , Song, M.J. , Kim, J. , Lee, B.H. , Loffler, F.E. et al. (2021) Cometabolic vinyl chloride degradation at acidic pH catalyzed by acidophilic methanotrophs isolated from alpine peat bogs. Environmental Science & Technology, 55, 5959–5969. 33843227 10.1021/acs.est.0c08766

[emi413156-bib-0016] Chu, K.H. & Alvarez‐Cohen, L. (1996) Trichloroethylene degradation by methane‐oxidizing cultures grown with various nitrogen sources. Water Environment Research, 68, 76–82.

[emi413156-bib-0017] Chu, K.‐H. & Alvarez‐Cohen, L. (1998) Effect of nitrogen source on growth and trichloroethylene degradation by methane‐oxidizing bacteria. Applied and Environmental Microbiology, 64, 3451–3457. 9726896 10.1128/aem.64.9.3451-3457.1998 PMC106746

[emi413156-bib-0018] Chu, K.‐H. & Alvarez‐Cohen, L. (1999) Evaluation of toxic effects of aeration and trichloroethylene oxidation on methanotrophic bacteria grown with different nitrogen sources. Applied and Environmental Microbiology, 65, 766–772. 9925614 10.1128/aem.65.2.766-772.1999 PMC91093

[emi413156-bib-0019] Crombie, A.T. & Murrell, J.C. (2014) Trace‐gas metabolic versatility of the facultative methanotroph *Methylocella silvestris* . Nature, 510, 148–151. 24776799 10.1038/nature13192

[emi413156-bib-0020] Danilova, O.V. , Kulichevskaya, I.S. , Rozova, O.N. , Detkova, E.N. , Bodelier, P.L. , Trotsenko, Y.A. et al. (2013) *Methylomonas paludis* sp. nov., the first acid‐tolerant member of the genus *Methylomonas*, from an acidic wetland. Journal of Systematics and Evolution, 63, 2282–2289. 10.1099/ijs.0.045658-0 23159751

[emi413156-bib-0021] Dawas‐Massalha, A. , Gur‐Reznik, S. , Lerman, S. , Sabbah, I. & Dosoretz, C.G. (2014) Co‐metabolic oxidation of pharmaceutical compounds by a nitrifying bacterial enrichment. Bioresource Technology, 167, 336–342. 24997377 10.1016/j.biortech.2014.06.003

[emi413156-bib-0022] Dedysh, S.N. (2009) Exploring methanotroph diversity in acidic northern wetlands: Molecular and cultivation‐based studies. Microbiology, 78, 655–669.

[emi413156-bib-0023] Dedysh, S.N. (2011) Cultivating uncultured bacteria from northern wetlands: Knowledge gained and remaining gaps. Frontiers in Microbiology, 2, 184. 21954394 10.3389/fmicb.2011.00184 PMC3174395

[emi413156-bib-0024] Dedysh, S.N. , Belova, S.E. , Bodelier, P.L. , Smirnova, K.V. , Khmelenina, V.N. , Chidthaisong, A. et al. (2007) *Methylocystis heyeri* sp. nov., a novel type II methanotrophic bacterium possessing ‘signature’ fatty acids of type I methanotrophs. Journal of Systematics and Evolution, 57, 472–479. 10.1099/ijs.0.64623-0 17329771

[emi413156-bib-0025] Dedysh, S.N. , Berestovskaya, Y.Y. , Vasylieva, L.V. , Belova, S.E. , Khmelenina, V.N. , Suzina, N.E. et al. (2004) *Methylocella tundrae* sp. nov., a novel methanotrophic bacterium from acidic tundra peatlands. Journal of Systematics and Evolution, 54, 151–156. 10.1099/ijs.0.02805-0 14742473

[emi413156-bib-0026] Dedysh, S.N. , Didriksen, A. , Danilova, O.V. , Belova, S.E. , Liebner, S. & Svenning, M.M. (2015) *Methylocapsa palsarum* sp. nov., a methanotroph isolated from a subArctic discontinuous permafrost ecosystem. Journal of Systematics and Evolution, 65, 3618–3624. 10.1099/ijsem.0.000465 26297585

[emi413156-bib-0027] Dedysh, S.N. & Dunfield, P.F. (2011) Facultative and obligate methanotrophs: How to identify and differentiate them. Methods in Enzymology, 495, 31–44. 21419913 10.1016/B978-0-12-386905-0.00003-6

[emi413156-bib-0028] Dedysh, S.N. , Dunfield, P.F. , Derakshani, M. , Stubner, S. , Heyer, J. & Liesack, W. (2003) Differential detection of type II methanotrophic bacteria in acidic peatlands using newly developed 16S rRNA‐targeted fluorescent oligonucleotide probes. FEMS Microbiology Ecology, 43, 299–308. 19719661 10.1111/j.1574-6941.2003.tb01070.x

[emi413156-bib-0029] Dedysh, S.N. , Khmelenina, V.N. , Suzina, N.E. , Trotsenko, Y.A. , Semrau, J.D. , Liesack, W. et al. (2002) *Methylocapsa acidiphila* gen. nov., sp. nov., a novel methane‐oxidizing and dinitrogen‐fixing acidophilic bacterium from Sphagnum bog. Journal of Systematics and Evolution, 52, 251–261. 10.1099/00207713-52-1-251 11837310

[emi413156-bib-0030] Dedysh, S.N. , Knief, C. & Dunfield, P.F. (2005) *Methylocella* species are facultatively methanotrophic. Journal of Bacteriology, 187, 4665–4670. 15968078 10.1128/JB.187.13.4665-4670.2005 PMC1151763

[emi413156-bib-0031] Dedysh, S.N. , Liesack, W. , Khmelenina, V.N. , Suzina, N.E. , Trotsenko, Y.A. , Semrau, J.D. et al. (2000) *Methylocella palustris* gen. nov., sp. nov., a new methane‐oxidizing acidophilic bacterium from peat bogs, representing a novel subtype of serine‐pathway methanotrophs. Journal of Systematics and Evolution, 50, 955–969. 10.1099/00207713-50-3-955 10843033

[emi413156-bib-0032] Dedysh, S.N. , Naumoff, D.G. , Vorobev, A.V. , Kyrpides, N. , Woyke, T. , Shapiro, N. et al. (2015) Draft genome sequence of *Methyloferula stellata* AR4, an obligate methanotroph possessing only a soluble methane monooxygenase. Genome Announcements, 3, e01555–e01514. 25745010 10.1128/genomeA.01555-14 PMC4358397

[emi413156-bib-0033] Dedysh, S.N. , Panikov, N.S. , Liesack, W. , Großkopf, R. , Zhou, J. & Tiedje, J.M. (1998) Isolation of acidophilic methane‐oxidizing bacteria from northern peat wetlands. Science, 282, 281–284. 9765151 10.1126/science.282.5387.281

[emi413156-bib-0034] Dedysh, S.N. , Panikov, N.S. & Tiedje, J.M. (1998) Acidophilic methanotrophic communities from sphagnum peat bogs. Applied and Environmental Microbiology, 64, 922–929. 9501432 10.1128/aem.64.3.922-929.1998 PMC106347

[emi413156-bib-0035] Denver, J.M. , Ator, S.W. , Fischer, J.M. , Harned, D.C. , Schubert, C. & Szabo, Z. (2014) In: U.S.G.S. Circular (Ed.) The quality of our nation's waters—Water quality in the Northern Atlantic Coastal Plain surficial aquifer system, Delaware, Maryland, New Jersey, New York, North Carolina, and Virginia, 1988–2009, p. 88. https://pubs.usgs.gov/circ/1353/ [Accessed 12th January 2022].

[emi413156-bib-0036] DeSimone, L.A. , McMahon, P.B. & Rosen, M.R. (2014) In: U.S.G.S. Circular (Ed.) The quality of our Nation's waters—Water quality in Principal Aquifers of the United States, 1991–2010, p. 151. https://pubs.usgs.gov/circ/1360/ [Accessed 12th January 2022].

[emi413156-bib-0037] Dopson, M. & Holmes, D.S. (2014) Metal resistance in acidophilic microorganisms and its significance for biotechnologies. Applied Microbiology and Biotechnology, 98, 8133–8144. 25104030 10.1007/s00253-014-5982-2

[emi413156-bib-0038] Dunfield, P.F. , Belova, S.E. , Vorob'ev, A.V. , Cornish, S.L. & Dedysh, S.N. (2010) *Methylocapsa aurea* sp. nov., a facultative methanotroph possessing a particulate methane monooxygenase, and emended description of the genus *Methylocapsa* . Journal of Systematics and Evolution, 60, 2659–2664. 10.1099/ijs.0.020149-0 20061505

[emi413156-bib-0039] Dunfield, P.F. & Dedysh, S.N. (2014) *Methylocella*: A gourmand among methanotrophs. Trends in Microbiology, 22, 368–369. 24874563 10.1016/j.tim.2014.05.004

[emi413156-bib-0040] Dunfield, P.F. , Khmelenina, V.N. , Suzina, N.E. , Trotsenko, Y.A. & Dedysh, S.N. (2003) *Methylocella silvestris* sp. nov., a novel methanotroph isolated from an acidic forest cambisol. Journal of Systematics and Evolution, 53, 1231–1239. 10.1099/ijs.0.02481-0 13130000

[emi413156-bib-0041] Eaddy, A. (2008) Scale‐up and characterization of an enrichment culture for bioaugmentation of the P‐Area chlorinated ethene plume at the Savannah River Site. Clemson, SC, USA: Clemson University.

[emi413156-bib-0042] EPA (2016) Fact sheet on trichloroethylene (TCE) . In. EPA (ed).

[emi413156-bib-0043] Esson, K.C. , Lin, X. , Kumaresan, D. , Chanton, J.P. , Murrell, J.C. & Kostka, J.E. (2016) Alpha‐ and gammaproteobacterial methanotrophs codominate the active methane‐oxidizing communities in an acidic boreal peat bog. Applied and Environmental Microbiology, 82, 2363–2371. 26873322 10.1128/AEM.03640-15 PMC4959502

[emi413156-bib-0044] Farhan Ul Haque, M. , Xu, H.J. , Murrell, J.C. & Crombie, A. (2020) Facultative methanotrophs—Diversity, genetics, molecular ecology and biotechnological potential: A mini‐review. Microbiology, 166, 894–908. 33085587 10.1099/mic.0.000977 PMC7660913

[emi413156-bib-0045] Fogel, M.M. , Taddeo, A.R. & Fogel, S. (1986) Biodegradation of chlorinated ethenes by a methane‐utilizing mixed culture. Applied and Environmental Microbiology, 51, 720–724. 3085587 10.1128/aem.51.4.720-724.1986 PMC238954

[emi413156-bib-0046] Fox, B.G. , Borneman, J.G. , Wackett, L.P. & Lipscomb, J.D. (1990) Haloalkene oxidation by the soluble methane monooxygenase from *Methylosinus trichosporium* OB3b: Mechanistic and environmental implications. Biochemistry, 29, 6419–6427. 2207083 10.1021/bi00479a013

[emi413156-bib-0047] Gertz, E.M. , Yu, Y.‐K. , Agarwala, R. , Schaffer, A.A. & Altschul, S.F. (2006) Composition‐based statistics and translated nucleotide searches: Improving the TBLASTN module of BLAST. BMC Biology, 4, 41. 17156431 10.1186/1741-7007-4-41 PMC1779365

[emi413156-bib-0048] Gesicka, A. , Oleskowicz‐Popiel, P. & Lezyk, M. (2021) Recent trends in methane to bioproduct conversion by methanotrophs. Biotechnology Advances, 53, 107861. 34710553 10.1016/j.biotechadv.2021.107861

[emi413156-bib-0049] Ghashghavi, M. , Jetten, M.S.M. & Lüke, C. (2017) Surveyof methanotrophic diversity in various ecosystems bydegenerate methane monooxygenase gene primers. AMB Express, 7, 162. 28831762 10.1186/s13568-017-0466-2 PMC5567572

[emi413156-bib-0050] Guerrero‐Cruz, S. , Vaksmaa, A. , Horn, M.A. , Niemann, H. , Pijuan, M. & Ho, A. (2021) Methanotrophs: Discoveries, environmental relevance, and a perspective on current and future applications. Frontiers in Microbiology, 12, 678057. 34054786 10.3389/fmicb.2021.678057 PMC8163242

[emi413156-bib-0051] Halsey, K.H. , Sayavedra‐Soto, L.A. , Bottomley, P.J. & Arp, D.J. (2005) Trichloroethylene degradation by butane‐oxidizing bacteria causes a spectrum of toxic effects. Applied Microbiology and Biotechnology, 68, 794–801. 15754184 10.1007/s00253-005-1944-z

[emi413156-bib-0052] Han, D. , Dedysh, S.N. & Liesack, W. (2018) Unusual genomic traits suggest *Methylocystis bryophila* S285 to be well adapted for life in peatlands. GBE, evy025, 623–628. 10.1093/gbe/evy025 PMC5808792 29390143

[emi413156-bib-0053] Hand, S. , Wang, B. & Chu, K.H. (2015) Biodegradation of 1,4‐dioxane: Effects of enzyme inducers and trichloroethylene. Science of the Total Environment, 520, 154–159. 25813968 10.1016/j.scitotenv.2015.03.031

[emi413156-bib-0054] Hanson, R.S.H. (1996) Methanotrophic bacteria. Microbiological Reviews, 60, 439–471. 8801441 10.1128/mr.60.2.439-471.1996 PMC239451

[emi413156-bib-0055] Hatzinger, P.B. , Banerjee, R. , Rezes, R. , Streger, S.H. , McClay, K. & Schaefer, C.E. (2017) Potential for cometabolic biodegradation of 1,4‐dioxane in aquifers with methane or ethane as primary substrates. Biodegradation, 28, 453–468. 29022194 10.1007/s10532-017-9808-7

[emi413156-bib-0056] Hatzinger, P.B. , Diebold, J. , Yates, C.A. & Cramer, R.J. (2006) Field demonstration of in situ perchlorate bioremediation in groundwater. Boston, MA: Springer.

[emi413156-bib-0057] Hazen, T.C. , Lombard, K.H. , Looney, B.B. , Enzien, M.V. , Dougherty, J.M. , Fliermans, C.B. et al. (1994) Summary of in situ bioremediation demonstration (methane biostimulation) via horizontal wells at the Savannah River Site Integrated Demonstration Project. In: Gee, G.W. & Wing, N.R. (Eds.) Proceedings of thirty‐third Hanford symposium on health and the environment: In‐situ remediation: Scientific basis for current and future technologies. Battelle: Columbus, pp. 135–150.

[emi413156-bib-0058] Hedegaard, M.J. , Schliemann‐Haug, M.A. , Milanovic, N. , Lee, C.O. , Boe‐Hansen, R. & Albrechtsen, H.‐J. (2020) Importance of methane oxidation for microbial degradation of the herbicide bentazone in drinking water production. Frontiers in Environmental Science, 8, 79.

[emi413156-bib-0059] Hesselsoe, M. , Boysen, S. , Iversen, N. , Jørgensen, L. , Murrell, J.C. , McDonald, I. et al. (2005) Degradation of organic pollutants by methane grown microbial consortia. Biodegradation, 16, 435–448. 15865157 10.1007/s10532-004-4721-2

[emi413156-bib-0060] Holmes, A.J. , Roslev, P. , McDonald, I.R. , Iversen, N. , Henriksen, K. & Murrell, J.C. (1999) Characterization of methanotrophic bacterial populations in soils showing atmospheric methane uptake. Applied and Environmental Microbiology, 65, 3312–3318. 10427012 10.1128/aem.65.8.3312-3318.1999 PMC91497

[emi413156-bib-0061] Hutchens, E. , Radajewski, S. , Dumont, M.G. , McDonald, I.R. & Murrell, J.C. (2004) Analysis of methanotrophic bacteria in Movile Cave by stable isotope probing. Environmental Microbiology, 6, 111–120. 14756876 10.1046/j.1462-2920.2003.00543.x

[emi413156-bib-0062] Im, J. & Semrau, J.D. (2011) Pollutant degradation by a *Methylocystis* strain SB2 grown on ethanol: Bioremediation via facultative methanotrophy. FEMS Microbiology Letters, 318, 137–142. 21362021 10.1111/j.1574-6968.2011.02249.x

[emi413156-bib-0063] Islam, T. , Torsvik, V. , Larsen, O. , Bodrossy, L. , Ovreas, L. & Birkeland, N.K. (2016) Acid‐tolerant moderately thermophilic methanotrophs of the class *Gammaproteobacteria* isolated from tropical topsoil with Methane Seeps. Frontiers in Microbiology, 7, 851. 27379029 10.3389/fmicb.2016.00851 PMC4908921

[emi413156-bib-0064] Jagadevan, S. & Semrau, J.D. (2013) Priority pollutant degradation by the facultative methanotroph, *Methylocystis* strain SB2. Applied Microbiology and Biotechnology, 97, 5089–5096. 22851017 10.1007/s00253-012-4310-y

[emi413156-bib-0065] Kalyuzhnaya, M.G. , Zabinsky, R. , Bowerman, S. , Baker, D.R. , Lidstrom, M.E. & Chistoserdova, L. (2006) Fluorescence in situ hybridization‐flow cytometry‐cell sorting‐based method for separation and enrichment of type I and type II methanotroph populations. Applied and Environmental Microbiology, 72, 4293–4301. 16751544 10.1128/AEM.00161-06 PMC1489643

[emi413156-bib-0501] Khider, M.L.K. , Brautaset, T. & Irla, M. (2021) Methane monooxygenases: central enzymes in methanotrophy with promising biotechnological applications. World Journal of Microbiology and Biotechnology, 37(4). Available from: 10.1007/s11274-021-03038-x PMC7994243 33765207

[emi413156-bib-0066] Kip, N. , Fritz, C. , Langelaan, E.S. , Pan, Y. , Bodrossy, L. , Pancotto, V. et al. (2012) Methanotrophic activity and diversity in different *Sphagnum magellanicum* dominated habitats in the southernmost peat bogs of Patagonia. Biogeosciences, 9, 47–55.

[emi413156-bib-0067] Kip, N. , Ouyang, W. , van Winden, J. , Raghoebarsing, A. , van Niftrik, L. , Pol, A. et al. (2011) Detection, isolation, and characterization of acidophilic methanotrophs from Sphagnum mosses. Applied and Environmental Microbiology, 77, 5643–5654. 21724892 10.1128/AEM.05017-11 PMC3165258

[emi413156-bib-0068] Knief, C. (2015) Diversity and habitat preferences of cultivated and uncultivated aerobic methanotrophic bacteria evaluated based on *pmoA* as molecular marker. Frontiers in Microbiology, 6, 1346. 26696968 10.3389/fmicb.2015.01346 PMC4678205

[emi413156-bib-0069] Kolb, S. & Horn, M.A. (2012) Microbial CH(4) and N(2)O consumption in acidic wetlands. Frontiers in Microbiology, 3, 78. 22403579 10.3389/fmicb.2012.00078 PMC3291872

[emi413156-bib-0070] Kox, M.A. , Haque, M.F.U. , van Alen, T.A. , Crombie, A.T. , Jetten, M.S. , den Camp, H.J.O. et al. (2019) Complete genome sequence of the aerobic facultative methanotroph *Methylocella tundrae* Strain T4. Microbiology Resource Announcements, 8, e00286–e00219. 31097502 10.1128/MRA.00286-19 PMC6522787

[emi413156-bib-0505] Kulikova, A.K. & Bezborodov, A.M. (2000) Oxidation of organic compounds by propane monooxygenase of Rhodococcus erythropolis 3/89. Applied Biochemistry and Microbiology, 36(3), 227–230. Available from: 10.1007/bf02742570 10867943

[emi413156-bib-0071] Lacroix, E. , Brovelli, A. , Barry, D.A. & Holliger, C. (2014) Use of silicate minerals for pH control during reductive dechlorination of chloroethenes in batch cultures of different microbial consortia. Applied and Environmental Microbiology, 80, 3858–3867. 24747895 10.1128/AEM.00493-14 PMC4054199

[emi413156-bib-0072] Lau, E. , Ahmad, A. , Steudler, P.A. & Cavanaugh, C.M. (2007) Molecular characterisation of methanotrophic communities in forest soils that consume atmospheric methane. FEMS Microbiology Ecology, 60, 490–500. 17391332 10.1111/j.1574-6941.2007.00308.x

[emi413156-bib-0073] Lawton, T.J. & Rosenzweig, A.C. (2016) Methane‐oxidizing enzymes: An upstream problem in biological gas‐to‐liquids conversion. Journal of the American Chemical Society, 138, 9327–9340. 27366961 10.1021/jacs.6b04568 PMC5242187

[emi413156-bib-0504] Lee, E.‐H. , Park, H. & Cho, K.‐S. (2011) Effect of substrate interaction on oxidation of methane and benzene in enriched microbial consortia from landfill cover soil. Journal of Environmental Science and Health, Part A, 46(9), 997–1007. Available from: 10.1080/10934529.2011.586266 21847790

[emi413156-bib-0074] Lee, H. , Baek, J.I. , Kim, S.J. , Kwon, K.K. , Rha, E. , Yeom, S.J. et al. (2020) Sensitive and rapid phenotyping of microbes with soluble methane monooxygenase using a droplet‐based assay. Frontiers in Bioengineering and Biotechnology, 8, 358. 32391352 10.3389/fbioe.2020.00358 PMC7193049

[emi413156-bib-0075] Lee, S.‐W. , Keeney, D.R. , Lim, D.‐H. , Dispirito, A.A. & Semrau, J.D. (2006) Mixed pollutant degradation by *Methylosinus trichosporium* OB3b expressing either soluble or particulate methane monooxygenase: Can the tortoise beat the hare? Applied and Environmental Microbiology, 72, 7503–7509. 17012599 10.1128/AEM.01604-06 PMC1694253

[emi413156-bib-0076] Leng, L. , Chang, J. , Geng, K. , Lu, Y. & Ma, K. (2015) Uncultivated *Methylocystis* species in paddy soil include facultative methanotrophs that utilize acetate. Microbial Ecology, 70, 88–96. 25475784 10.1007/s00248-014-0540-0

[emi413156-bib-0077] Little, C.D. , Palumbo, A.V. , Herbes, S.E. , Lidstrom, M.E. , Tyndall, R.L. & Gilmer, P.J. (1988) Trichloroethylene biodegradation by a methane‐oxidizing bacterium. Applied and Environmental Microbiology, 54, 951–956. 16347616 10.1128/aem.54.4.951-956.1988 PMC202578

[emi413156-bib-0078] Lontoh, S. & Semrau, J.D. (1998) Methane and trichloroethylene degradation by *Methylosinus trichosporium* OB3b expressing particulate methane monooxygenase. Applied and Environmental Microbiology, 64, 1106–1114. 16349516 10.1128/aem.64.3.1106-1114.1998 PMC106375

[emi413156-bib-0079] Madigan, M.T. (2018) Brock biology of microorganisms. San Francisco: Benjamin Cummings.

[emi413156-bib-0080] Mahendra, S. & Alvarez‐Cohen, L. (2006) Kinetics of 1,4‐Dioxane biodegradation by monooxygenase‐expressing bacteria. Environmental Science & Technology, 40, 5435–5442. 16999122 10.1021/es060714v

[emi413156-bib-0081] Malachowsky, K.J. , Phelps, T.J. , Teboli, A.B. , Minnikin, D.E. & White, D.C. (1994) Aerobic mineralization of trichloroethylene, vinyl chloride, and aromatic compounds by *Rhodococcus* species. Applied and Environmental Microbiology, 60, 542–548. 16349184 10.1128/aem.60.2.542-548.1994 PMC201346

[emi413156-bib-0082] Mangold, S. , Rao Jonna, V. & Dopson, M. (2013) Response of *Acidithiobacillus caldus* toward suboptimal pH conditions. Extremophiles, 17, 689–696. 23712908 10.1007/s00792-013-0553-5

[emi413156-bib-0508] McCarty, P.L. , Chu, M.‐Y. & Kitanidis, P.K. (2007) Electron donor and pH relationships for biologically enhanced dissolution of chlorinated solvent DNAPL in groundwater. European Journal of Soil Biology, 43(5–6), 276–282. Available from: 10.1016/j.ejsobi.2007.03.004

[emi413156-bib-0083] McDonald, I.R. , Hall, G.H. , Pickup, R.W. & Colin Murrell, J. (1996) Methane oxidation potential and preliminary analysis of methanotrophs in blanket bog peat using molecular ecology techniques. FEMS Microbiology Ecology, 21, 197–211.

[emi413156-bib-0084] McDonald, I.R. , Uchiyama, H. , Kambe, S. , Yagi, O. & Murrell, J.C. (1997) The soluble methane monooxygenase gene cluster of the trichloroethylene‐degrading methanotroph *Methylocystis* sp. strain M. Applied and Environmental Microbiology, 63, 1898–1904. 9143121 10.1128/aem.63.5.1898-1904.1997 PMC168481

[emi413156-bib-0085] Mills, J.C., IV , Wilson, J.T. , Wilson, B.H. , Weidemier, T.H. & Freedman, D.L. (2018) Quantification of TCE co‐oxidation in groundwater using a 14C‐assay. Groundwater Management & Regulation, 38, 57–67.

[emi413156-bib-0086] Miroshnikov, K.K. , Didriksen, A. , Naumoff, D.G. , Huntemann, M. , Clum, A. , Pillay, M. et al. (2017) Draft genome sequence of *Methylocapsa palsarum* NE2(T), an obligate methanotroph from subarctic soil. Genome Announcements, 5, e00504–e00517. 28619793 10.1128/genomeA.00504-17 PMC5473262

[emi413156-bib-0087] Mohammadi, S. , Pol, A. , van Alen, T.A. , Jetten, M.S. & Op den Camp, H.J. (2017) *Methylacidiphilum fumariolicum* SolV, a thermoacidophilic ‘Knallgas’ methanotroph with both an oxygen‐sensitive and ‐insensitive hydrogenase. The ISME Journal, 11, 945–958. 27935590 10.1038/ismej.2016.171 PMC5364354

[emi413156-bib-0088] Mohammadi, S.S. , Schmitz, R.A. , Pol, A. , Berben, T. , Jetten, M.S.M. & Op den Camp, H.J.M. (2019) The acidophilic methanotroph *Methylacidimicrobium tartarophylax* 4AC grows as autotroph on H2 under microoxic conditions. Frontiers in Microbiology, 10, 2352. 31681216 10.3389/fmicb.2019.02352 PMC6813726

[emi413156-bib-0089] Nazaries, L. , Murrell, J.C. , Millard, P. , Baggs, L. & Singh, B.K. (2013) Methane, microbes and models: Fundamental understanding of the soil methane cycle for future predictions. Environmental Microbiology, 15, 2395–2417. 23718889 10.1111/1462-2920.12149

[emi413156-bib-0090] Nguyen, N.L. , Yu, W.J. , Gwak, J.H. , Kim, S.J. , Park, S.J. , Herbold, C.W. et al. (2018) Genomic insights Into the acid adaptation of novel methanotrophs enriched from acidic forest soils. Frontiers in Microbiology, 9, 1982. 30210468 10.3389/fmicb.2018.01982 PMC6119699

[emi413156-bib-0091] Nordstrom, D.K. , Alpers, C.N. , Ptacek, C.J. & Blowes, D.W. (2000) Negative pH and extremely acidic mine waters from Iron Mountain, California. Environmental Science & Technology, 34, 254–258.

[emi413156-bib-0503] Oldenhuis, R. , Vink, R.L. , Janssen, D.B. & Witholt, B. (1989) Degradation of chlorinated aliphatic hydrocarbons by Methylosinus trichosporium OB3b expressing soluble methane monooxygenase. Applied and Environmental Microbiology, 55(11), 2819–2826. Available from: 10.1128/aem.55.11.2819-2826.1989 2624462 PMC203175

[emi413156-bib-0092] Oldenhuis, R. , Oedzes, J.Y. , Van der Waarde, J. & Janssen, D.B. (1991) Kinetics of chlorinated hydrocarbon degradation by *Methylosinus trichosporium* OB3b and toxicity of trichloroethylene. Applied and Environmental Microbiology, 57, 7–14. 2036023 10.1128/aem.57.1.7-14.1991 PMC182657

[emi413156-bib-0093] Op den Camp, H.J. , Islam, T. , Stott, M.B. , Harhangi, H.R. , Hynes, A. , Schouten, S. et al. (2009) Environmental, genomic and taxonomic perspectives on methanotrophic *Verrucomicrobia* . Environmental Microbiology Reports, 1, 293–306. 23765882 10.1111/j.1758-2229.2009.00022.x

[emi413156-bib-0094] Oremland, R.S. & Culbertson, C.W. (1992) Importance of methane‐oxidizing bacteria in the methane budget as revealed by the use of a specific inhibitor. Nature, 356, 421–423.

[emi413156-bib-0095] Oshkin, I.Y. , Miroshnikov, K.K. & Dedysh, S.N. (2019) Draft genome sequence of *Methylocystis heyeri* H2(T), a methanotroph with habitat‐specific adaptations, isolated from a peatland ecosystem. Microbiology Resource Announcements, 8, e00454–e00419. 31320431 10.1128/MRA.00454-19 PMC6639610

[emi413156-bib-0096] Papadopoulou, A. , Hedegaard, M.J. , Dechesne, A. , Albrechtsen, H.J. , Musovic, S. & Smets, B.F. (2019) Methanotrophic contribution to biodegradation of phenoxy acids in cultures enriched from a groundwater‐fed rapid sand filter. Applied Microbiology and Biotechnology, 103, 1007–1019. 30474728 10.1007/s00253-018-9501-8

[emi413156-bib-0097] Picone, N. , Mohammadi, S.S. , Waajen, A.C. , van Alen, T.A. , Jetten, M.S.M. , Pol, A. et al. (2020) More than a methanotroph: A broader substrate spectrum for *Methylacidiphilum fumariolicum* SolV. Frontiers in Microbiology, 11, 604485. 33381099 10.3389/fmicb.2020.604485 PMC7768010

[emi413156-bib-0098] Pol, A. , Heijmans, K. , Harhangi, H.R. , Tedesco, D. , Jetten, M.S. & Op den Camp, H.J. (2007) Methanotrophy below pH 1 by a new Verrucomicrobia species. Nature, 450, 874–878. 18004305 10.1038/nature06222

[emi413156-bib-0507] Puentes Jácome, L.A. , Wang, P.‐H. , Molenda, O. , Li, Y.X. , (Jine‐J.), Islam, M.A. , & Edwards, E.A. (2019) Sustained dechlorination of vinyl chloride to ethene in dehalococcoides‐enriched cultures grown without addition of exogenous vitamins and at low ph. Environmental Science & Technology, 53(19), 11364–11374. Available from: 10.1021/acs.est.9b02339 31441646

[emi413156-bib-0099] Rahman, M.T. , Crombie, A. , Moussard, H. , Chen, Y. & Murrell, J.C. (2011) Acetate repression of methane oxidation by supplemental *Methylocella silvestris* in a peat soil microcosm. Applied and Environmental Microbiology, 77, 4234–4236. 21515721 10.1128/AEM.02902-10 PMC3131658

[emi413156-bib-0100] Redmond, M.C. , Valentine, D.L. & Sessions, A.L. (2010) Identification of novel methane‐, ethane‐, and propane‐oxidizing bacteria at marine hydrocarbon seeps by stable isotope probing. Applied and Environmental Microbiology, 76, 6412–6422. 20675448 10.1128/AEM.00271-10 PMC2950463

[emi413156-bib-0101] Ricke, P. , Kube, M. , Nakagawa, S. , Erkel, C. , Reinhardt, R. & Liesack, W. (2005) First genome data from uncultured upland soil cluster alpha methanotrophs provide further evidence for a close phylogenetic relationship to *Methylocapsa acidiphila* B2 and for high‐affinity methanotrophy involving particulate methane monooxygenase. Applied and Environmental Microbiology, 71, 7472–7482. 16269789 10.1128/AEM.71.11.7472-7482.2005 PMC1287704

[emi413156-bib-0102] Rowlands, D. (2004) *Development of optimal pH for degradation of chlorinated solvents by the KB‐1 anaerobic bacterial culture*: Prepared for Geosyntec Consultants/SiREM.

[emi413156-bib-0502] Salter, A.J. , Johnson, R. & Tratnyek Paul, G. (2010) Degradation of 1, 2, 3‐ trichloropropane by zero‐valent zinc: Laboratory assessment for field application, Proceedings of the 7th International Conference on Remediation of Chlorinated and Recalcitrant Compounds. CA: Monterey.

[emi413156-bib-0103] Samin, G. & Janssen, D.B. (2012) Transformation and biodegradation of 1,2,3‐trichloropropane (TCP). Environmental Science and Pollution Research, 19, 3067–3078. 22875418 10.1007/s11356-012-0859-3 PMC3414701

[emi413156-bib-0104] Schaefer, C.E. , Lippincott, D.R. & Steffan, R.J. (2010) Field‐scale evaluation of bioaugmentation dosage for treating chlorinated ethenes. Groundwater Management & Regulation, 30, 113–124.

[emi413156-bib-0105] Schäfer, D. , Köber, R. & Dahmke, A. (2003) Competing TCE and *cis*‐DCE degradation kinetics by zero‐valent iron—Experimental results and numerical simulation. Journal of Contaminant Hydrology, 65, 183–202. 12935949 10.1016/S0169-7722(02)00239-5

[emi413156-bib-0106] Schmitz, R.A. , Peeters, S.H. , Versantvoort, W. , Picone, N. , Pol, A. , Jetten, M.S.M. et al. (2021) Verrucomicrobial methanotrophs: Ecophysiology of metabolically versatile acidophiles. FEMS Microbiology Reviews, 45, fuab007. 10.1093/femsre/fuab007 PMC8498564 33524112

[emi413156-bib-0107] Semprini, L. , Roberts, P.V. , Hopkins, G.D. & McCarty, P.L. (1990) A field evaluation of in‐situ biodegradation of chlorinated ethenes: Part 2, results of biostimulation and biotransformation experiments. Groundwater, 28, 715–727.

[emi413156-bib-0108] Semrau, J.D. (2011) Bioremediation via methanotrophy: Overview of recent findings and suggestions for future research. Frontiers in Microbiology, 2, 209. 22016748 10.3389/fmicb.2011.00209 PMC3191459

[emi413156-bib-0109] Shao, Y. , Hatzinger, P.B. , Streger, S.H. , Rezes, R.T. & Chu, K.‐H. (2019) Evaluation of methanotrophic bacterial communities capable of biodegrading trichloroethene (TCE) in acidic aquifers. Biodegradation, 30, 173–190. 30989421 10.1007/s10532-019-09875-w

[emi413156-bib-0110] Sharma, A. , Parashar, D. & Satyanarayana, T. (2016) Acidophilic microbes: Biology and applications. In: Rampelotto, P.H. (Ed.) Biotechnology of extremophiles, grand challenges in biology and biotechnology, Vol. 1. Switzerland: Springer International Publishing, pp. 215–241.

[emi413156-bib-0111] Sharp, J.O. , Wood, T.K. & Alvarez‐Cohen, L. (2005) Aerobic biodegradation of *N*‐nitrosodimethylamine (NDMA) by axenic bacterial strains. Biotechnology and Bioengineering, 89, 608–618. 15672376 10.1002/bit.20405

[emi413156-bib-0112] Shigematsu, T. , Hanada, S. , Eguchi, M. , Kamagata, Y. , Kanagawa, T. & Kurane, R. (1999) Soluble methane monooxygenase gene clusters from trichloroethylene‐degrading *Methylomonas* sp. strains and detection of methanotrophs during in situ bioremediation. Applied and Environmental Microbiology, 65, 5198–5206. 10583965 10.1128/aem.65.12.5198-5206.1999 PMC91705

[emi413156-bib-0113] Shukla, A.K. , Vishwakarma, P. , Upadhyay, S. , Tripathi, A.K. , Prasana, H. & Dubey, S.K. (2009) Biodegradation of trichloroethylene (TCE) by methanotrophic community. Bioresource Technology, 100, 2469–2474. 19157866 10.1016/j.biortech.2008.12.022

[emi413156-bib-0114] Siljanen, H.M. , Saari, A. , Bodrossy, L. & Martikainen, P.J. (2012) Seasonal variation in the function and diversity of methanotrophs in the littoral wetland of a boreal eutrophic lake. FEMS Microbiology Ecology, 80, 548–555. 22296339 10.1111/j.1574-6941.2012.01321.x

[emi413156-bib-0115] Singh, J.S. & Singh, D.P. (2017) Methanotrophs: An emerging bioremediation tool with unique broad spectrum methane monooxygenase (MMO) enzyme. In: Singh, J. & Seneviratne, G. (Eds.) Agro‐environmental sustainability. Cham: Springer.

[emi413156-bib-0116] Steffan, R.J. , McClay, K. , Vainberg, S. , Condee, C.W. & Zhang, D. (1997) Biodegradation of the gasoline oxygenates methyl tert‐butyl ether, ethyl tert‐butyl ether, and tert‐amyl methyl ether by propane‐oxidizing bacteria. Applied and Environmental Microbiology, 63, 4216–4222. 9361407 10.1128/aem.63.11.4216-4222.1997 PMC168740

[emi413156-bib-0117] Steffan, R.J. & Vainberg, S. (2013) Production and handling of *Dehalococcoides* bioaugmentation cultures. In: Stroo, H.F. , Leeson, A. & Ward, C.W. (Eds.) Bioaugmentation for groundwater remediation. New York, NY, USA: Springer.

[emi413156-bib-0118] Stoecker, K. , Bendinger, B. , Schoning, B. , Nielsen, P.H. , Nielsen, J.L. , Baranyi, C. et al. (2006) Cohn's *Crenothrix* is a filamentous methane oxidizer with an unusual methane monooxygenase. Proceedings of the National Academy of Sciences of the United States of America, 103, 2363–2367. 16452171 10.1073/pnas.0506361103 PMC1413686

[emi413156-bib-0119] Strong, P.J. , Xie, S. & Clarke, W.P. (2015) Methane as a resource: Can the methanotrophs add value? Environmental Science & Technology, 49, 4001–4018. 25723373 10.1021/es504242n

[emi413156-bib-0120] Stroo, H.F. & Ward, C.H.E. (2010) In situ remediation of chlorinated solvent plumes .

[emi413156-bib-0121] Szwast, N.A. (2021) Evaluation of methanotrophic biodegradation of TCE at Low pH. Clemson University. All Theses. 3625. https://tigerprints.clemson.edu/all_theses/3625 [Accessed 12th January 2022].

[emi413156-bib-0122] Tamas, I. , Smirnova, A.V. , He, Z. & Dunfield, P.F. (2014) The (d)evolution of methanotrophy in the *Beijerinckiaceae*—A comparative genomics analysis. The ISME Journal, 8, 369–382. 23985741 10.1038/ismej.2013.145 PMC3906808

[emi413156-bib-0123] Theisen, A.R. , Ali, M.H. , Radajewski, S. , Dumont, M.G. , Dunfield, P.F. , McDonald, I.R. et al. (2005) Regulation of methane oxidation in the facultative methanotroph *Methylocella silvestris* BL2. Molecular Microbiology, 58, 682–692. 16238619 10.1111/j.1365-2958.2005.04861.x

[emi413156-bib-0124] Tsien, H.‐C. , Brusseau, G.A. , Hanson, R.S. & Waclett, L. (1989) Biodegradation of trichloroethylene by *Methylosinus trichosporium* OB3b. Applied and Environmental Microbiology, 55, 3155–3161. 2515801 10.1128/aem.55.12.3155-3161.1989 PMC203239

[emi413156-bib-0125] UniProt Consortium . (2021) UniProt: The universal protein knowledgebase in 2021. Nucleic Acids Research, 49(D1), D480–D489. 33237286 10.1093/nar/gkaa1100 PMC7778908

[emi413156-bib-0126] Vainberg, S. , Condee, C.W. & Steffan, R.J. (2009) Large‐scale production of bacterial consortia for remediation of chlorinated solvent‐contaminated groundwater. Journal of Industrial Microbiology & Biotechnology, 36, 1189–1197. 19521729 10.1007/s10295-009-0600-5

[emi413156-bib-0127] van Teeseling, M.C. , Pol, A. , Harhangi, H.R. , van der Zwart, S. , Jetten, M.S. , Op den Camp, H.J. et al. (2014) Expanding the verrucomicrobial methanotrophic world: Description of three novel species of *Methylacidimicrobium* gen. nov. Applied and Environmental Microbiology, 80, 6782–6791. 25172849 10.1128/AEM.01838-14 PMC4249049

[emi413156-bib-0128] Vekeman, B. , Kerckhof, F.M. , Cremers, G. , de Vos, P. , Vandamme, P. , Boon, N. et al. (2016) New *Methyloceanibacter* diversity from North Sea sediments includes methanotroph containing solely the soluble methane monooxygenase. Environmental Microbiology, 18, 4523–4536. 27501305 10.1111/1462-2920.13485

[emi413156-bib-0129] Vorobev, A.V. , Baani, M. , Doronina, N.V. , Brady, A.L. , Liesack, W. , Dunfield, P.F. et al. (2011) *Methyloferula stellata* gen. nov., sp. nov., an acidophilic, obligately methanotrophic bacterium that possesses only a soluble methane monooxygenase. Journal of Systematics and Evolution, 61, 2456–2463. 10.1099/ijs.0.028118-0 21097638

[emi413156-bib-0130] Wackett, L.P. , Brusseau, G.A. , Householder, S.R. & Hanson, R.S. (1989) Survey of microbial oxygenases: Trichloroethylene degradation by propane‐oxidizing bacteria. Applied and Environmental Microbiology, 55, 2960–2964. 2624467 10.1128/aem.55.11.2960-2964.1989 PMC203198

[emi413156-bib-0131] Wang, B. & Chu, K.‐H. (2017) Cometabolic biodegradation of 1,2,3‐trichloropropane by propane‐oxidizing bacteria. Chemosphere, 168, 1494–1497. 27939660 10.1016/j.chemosphere.2016.12.007

[emi413156-bib-0506] Wilkins, P.C. , Dalton, H. , Samuel, C.J. & Green, J. (1994) Further evidence for multiple pathways in soluble methane‐monooxygenase‐catalysed oxidations from the measurement of deuterium kinetic isotope effects. European Journal of Biochemistry, 226(2), 555–560. Available from: 10.1111/j.1432-1033.1994.tb20080.x 8001570

[emi413156-bib-0132] Wilson, J.T. & Wilson, B.H. (1985) Biotransformation of trichloroethylene in soil. Applied and Environmental Microbiology, 49, 242–243. 3919642 10.1128/aem.49.1.242-243.1985 PMC238380

[emi413156-bib-0133] Wüst, W.F. , Köber, R. , Schlicker, O. & Dahmke, A. (1999) Combined zero‐and first‐order kinetic model of the degradation of TCE and *cis*‐DCE with commercial iron. Environmental Science & Technology, 33, 4304–4309.

[emi413156-bib-0134] Yang, Y. (2012) Exploring anaerobic reductive dechlorination at low pH environments. Knoxville, TN, USA: University of Tennessee.

[emi413156-bib-0135] Yang, Y. , Capiro, N.L. & Marcet, T.F. (2017) Organohalide respiration with chlorinated ethenes under low pH conditions. Environmental Science & Technology, 51, 8579–8588. 28665587 10.1021/acs.est.7b01510

